# Impact of molecular diagnostic tests on diagnostic and treatment delays in tuberculosis: a systematic review and meta-analysis

**DOI:** 10.1186/s12879-022-07855-9

**Published:** 2022-12-14

**Authors:** Jae Hyoung Lee, Tushar Garg, Jungsil Lee, Sean McGrath, Lori Rosman, Samuel G. Schumacher, Andrea Benedetti, Zhi Zhen Qin, Genevieve Gore, Madhukar Pai, Hojoon Sohn

**Affiliations:** 1 Department of Pediatrics, Johns Hopkins University School of Medicine, Baltimore, USA; 2 Department of Epidemiology, Johns Hopkins Bloomberg School of Public Health, Baltimore, USA; 3 London School of Hygiene & Tropical Medicine, London, UK; 4 Department of Biostatistics, Harvard T.H. Chan School of Public Health, Boston, USA; 5 Welch Medical Library, John Hopkins University School of Medicine, Baltimore, USA; 6 Foundation for Innovative New Diagnostics, Geneva, Switzerland; 7 Department of Epidemiology, Biostatistics and Occupational Health, McGill University, Montreal, Canada; 8 Respiratory Epidemiology & Clinical Research Unit, McGill University Health Centre, Montreal, Canada; 9 Stop TB Partnership, Geneva, Switzerland; 10 Schulich Library of Physical Sciences, Life Sciences, and Engineering, McGill University, Montreal, Canada; 11 McGill International TB Centre, McGill University, Montreal, Canada; 12 Department of Preventive Medicine, College of Medicine, Seoul National University, Seoul, South Korea

**Keywords:** Nucleic acid amplification tests, Communicable diseases, Point-of-Care Systems, Global Health

## Abstract

**Background:**

Countries with high TB burden have expanded access to molecular diagnostic tests. However, their impact on reducing delays in TB diagnosis and treatment has not been assessed. Our primary aim was to summarize the quantitative evidence on the impact of nucleic acid amplification tests (NAAT) on diagnostic and treatment delays compared to that of the standard of care for drug-sensitive and drug-resistant tuberculosis (DS-TB and DR-TB).

**Methods:**

We searched MEDLINE, EMBASE, Web of Science, and the Global Health databases (from their inception to October 12, 2020) and extracted time delay data for each test. We then analysed the diagnostic and treatment initiation delay separately for DS-TB and DR-TB by comparing smear vs Xpert for DS-TB and culture drug sensitivity testing (DST) vs line probe assay (LPA) for DR-TB. We conducted random effects meta-analyses of differences of the medians to quantify the difference in diagnostic and treatment initiation delay, and we investigated heterogeneity in effect estimates based on the period the test was used in, empiric treatment rate, HIV prevalence, healthcare level, and study design. We also evaluated methodological differences in assessing time delays.

**Results:**

A total of 45 studies were included in this review (DS = 26; DR = 20). We found considerable heterogeneity in the definition and reporting of time delays across the studies. For DS-TB, the use of Xpert reduced diagnostic delay by 1.79 days (95% CI − 0.27 to 3.85) and treatment initiation delay by 2.55 days (95% CI 0.54–4.56) in comparison to sputum microscopy. For DR-TB, use of LPAs reduced diagnostic delay by 40.09 days (95% CI 26.82–53.37) and treatment initiation delay by 45.32 days (95% CI 30.27–60.37) in comparison to any culture DST methods.

**Conclusions:**

Our findings indicate that the use of World Health Organization recommended diagnostics for TB reduced delays in diagnosing and initiating TB treatment. Future studies evaluating performance and impact of diagnostics should consider reporting time delay estimates based on the standardized reporting framework.

**Supplementary Information:**

The online version contains supplementary material available at 10.1186/s12879-022-07855-9.

## Introduction

In the last two decades, there has been a global push to end the tuberculosis (TB) epidemic by setting aggressive targets with the End TB Strategy [1]. Nonetheless, in 2020, there were an estimated 9.9 million TB cases and 1.3 million deaths, of which an estimated 40% went undiagnosed [2]. These missed diagnoses, made worse by the ongoing COVID-19 pandemic, perpetuate transmission and present significant challenges in ending TB [2]. Implementing diagnostic tools that improve detection and reduce diagnostic and treatment delays is critical in overcoming these gaps in TB care [3, 4].

GeneXpert MTB/RIF® and MTB/RIF Ultra® (Xpert) and line probe assays (LPA) are commercial nucleic acid amplification tests (NAATs) that have good diagnostic accuracy with the capacity to diagnose drug sensitive (DS-TB) and drug resistant TB (DR-TB) within 1–2 days of sample processing [5, 6]. Anticipating improvements in accurate and timely TB diagnosis, these NAATs were recommended by the World Health Organization (WHO) [7, 8]. Since then, unprecedented efforts have been made by National Tuberculosis Programs (NTPs) across the globe to scale up these tests and included them as part of the routine TB diagnostic algorithms [9–11]. These NAATs have proven to have high accuracy, and research has increasingly focused on studying their actual clinical impact [10, 12–16]. While there are systematic reviews on the diagnostic accuracy of Xpert and LPAs [6, 17, 18], and others that separately describe diagnostic and treatment delays experienced by TB patients [19], no study has summarized the impact of NAATs on reducing time delays in diagnosis and treatment of TB.

Therefore, the main objective of our systematic review was to summarize the available quantitative evidence on the impact of NAATs on diagnostic and treatment delays compared to that of the standard of care for DS-TB and DR-TB. As the secondary objective, we investigated the potential sources of heterogeneity on the effect estimates, including the period the tests were used (pre-2015, post 2015), empiric treatment rate, HIV prevalence, healthcare level, and type of study design (randomized controlled trial, observational study design). We also describe methodological areas of concern in assessing time delays, an aspect that has not been adequately addressed in previous systematic reviews of diagnostic delays in TB.

## Methods

### Study selection criteria and operational definitions

**Fig. 1:**
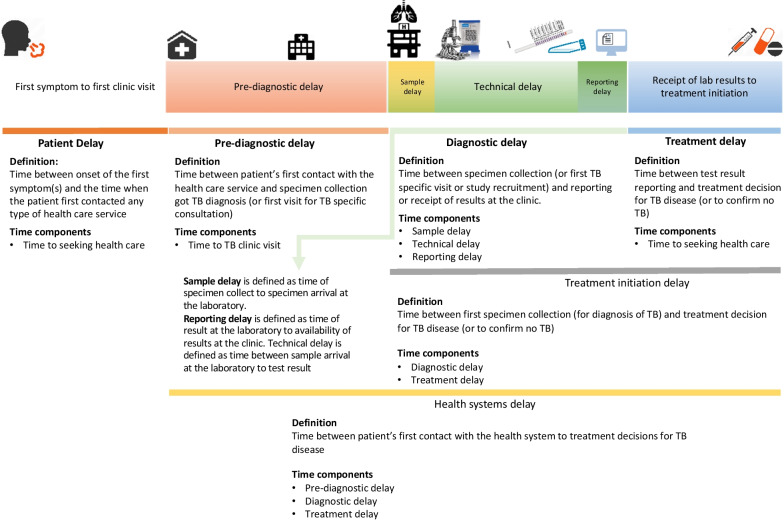
Conceptual framework of time delay components in diagnosis and treatment of tuberculosis. The illustrations depicted here are our own

Prior to the review, we developed a conceptual framework for classification of essential time delay components and definitions [20, 21] (Fig. 1). This framework standardized time delays and provided structural guidance in assessing time delays reported in the studies included in this review. We defined *diagnostic delay* as the time between initial patient contact with a clinic or sputum collection to reporting of results. *Treatment delay* was defined as the time between results and initiation of anti-TB treatment. And the combination of diagnostic delay and treatment delay was referred to as *treatment initiation delay*.

Our review focused on the impact of the World Health Organization (WHO)-recommended rapid diagnostics (WRD), specifically Xpert® MTB/RIF and MTB/RIF Ultra assay (Xpert) and GenoType MTBDR*plus* and Inno-LiPA RifTB (both referred to as LPA here on), because of their rapid uptake at the global level [2]. Several other tests have been recommended since 2020, but we did not include them in our systematic review because data is still limited [22].

We included only peer-reviewed studies that assessed time delays in the process of diagnosis and treatment of DS-TB and DR-TB with the index test as NAAT and a respective comparator test (e.g., smear for Xpert and culture DST for LPAs). We did not restrict our studies based on geography, settings, language, or type of study design. We excluded studies if they: (1) did not include primary data; (2) did not report all data necessary for meta-analysis; (3) were reviews or modelling studies; (4) only reported ‘run-time’ or turnaround time of the test (e.g., “2 h to run” Xpert test); and (5) focused on childhood or extra-pulmonary TB. For conference abstracts, we contacted the authors to see if there was a manuscript in preparation to obtain relevant data. Similarly, we requested original data from the authors when a study did not report time delay estimates as per our study requirements.

### Study search strategy, study selection, and data extraction

The present systematic review is an update to the systematic review published in the lead author’s (HS) doctoral thesis in 2016 [23]. The original and updated search were undertaken on January 31, 2015, and October 12, 2020, respectively. We identified eligible studies from MEDLINE, EMBASE, Web of Science, and the Global Health databases that included terms associated with time, like “delay” and “time to treatment” (see Additional file 1 for the complete search strategy). We also consulted references of included articles and previous systematic reviews focusing on the diagnostic accuracy of NAATs, and experts in the fields of TB diagnostics to identify additional studies not included in the database search. After removing duplicates, two reviewers (SGC, ZZQ, or HS—original review; JSL, JHL, or TG—updated review) independently screened titles and abstracts, followed by full-text review for inclusion (HS, SGS—original review; JSL, JHL—updated review). Any discrepancies were resolved by consensus or, in case of the updated review, a third reviewer (HS, TG).

Google Forms (Google LLC, Mountain View, CA, USA) was used for the initial review, but in the updated review, this data was incorporated into Covidence (Veritas Health Innovation, Melbourne, Australia) to manage the review and extract data [24]. The data extraction tools were pilot tested, using five studies in the full text review pool, prior to conducting full data extraction. A set of reviewers (HS—original review; JL, JHL—updated review) extracted the data before it was examined by separate reviewers (SGS—original review, TG—updated review) to resolve any discrepancies in the extracted data. We extracted data on study design, geographic setting, operational context, time delays for both the index and comparator tests, and delay definitions. Units of time were converted into the number of days. An example data extraction tool is available in Additional file 3.

### Quality assessment of time delay estimates

Unlike quality assessment tools for diagnostic accuracy studies, there is currently no established method or checklist that can be used to assess the quality of studies investigating time delays or time to event study outcomes [25]. Therefore, we developed a matrix of key methodologic and contextual information necessary to determine the usefulness and comparability of the time delay reported. These included (1) provision of a clear definition of measuring time delay and reporting the time delay estimates (“delay definition”); (2) use of appropriate statistical methods to report and assess changes in time delays (“statistical methods”); (3) evaluating time estimates alongside patient-important outcomes (“patient important outcomes”), which included culture conversion, TB treatment outcomes, infection control and/or contact tracing.

The provision of a clear delay definition was a binary variable with “Yes” and “No” options, where “Yes” indicated that the time delay term was defined clearly indicating its start and end time points with the delay estimate. The other two quality indicators were ranked on a high–medium–low scale. For the statistical method assessment, high quality studies evaluated the distribution of time delay and whether it used proper statistical methods [randomized controlled trial (RCT) or propensity score method for observational studies] that adjust estimates for proper comparison with a measure of variance to assess time delays between the index and the comparator test. Medium-quality studies evaluated the distribution of time delay with uncertainty estimates but did not use appropriate statistical methods for comparative assessment of time delays. And low-quality studies neither evaluated the distribution nor compared the time delay. For patient-important outcomes, high-quality studies analysed the relative risk or odds of improvement in culture conversion with the amount of time saved in TB treatment initiation. Medium quality studies reported time estimate alongside patient-important outcomes but without direct analysis, and low-quality studies did not consider patient-important outcomes at all.

### Data synthesis and meta-analysis

We calculated overall medians and IQRs of diagnostic and treatment initiation delay for each diagnostic test (Xpert vs. smear, LPA vs. any culture DST methods) from the medians and means reported by the individual studies. Additionally, using the extracted raw data, we applied the Mann–Whitney U test on overall medians to determine the statistical significance of the median time estimates between the index and comparator tests. We assumed no confounding in the primary studies.

We then conducted a meta-analysis using the quantile estimation (QE) method developed by McGrath et al. to assess the absolute reduction in diagnostic and treatment initiation delay using NAATs [26]. The method involves estimating the variance of the difference of medians of each study and pooling them using the standard inverse variance method. Time to event data are non-normally distributed variables that are primarily reported in medians and IQRs. As units of delay measurements (days) were uniform across all studies, the effect size was chosen to be the raw difference of medians in time delay for both diagnostic and treatment initiation delays. We used a random effects model because the studies differed importantly in characteristics that may lead to variations in the effect size [27, 28]. Between-study heterogeneity was estimated by the method of restricted maximum likelihood. Since this method requires complete data from median (or mean), IQR (or SD), and sample size, studies that did not report all the data points were excluded for the analysis.

Given the multifactorial nature of the studies, we also evaluated the heterogeneity based on the I-squared statistic, where a value greater than 75% is considered to be considerably heterogeneous [28, 29]. We conducted subgroup analyses to identify possible sources of heterogeneity and to assess key factors (pre-2015 vs. post-2015, RCT vs. observational, etc.) that can variably influence the magnitude of our effect size estimate. We specifically chose 2015 as our cut-off time point not only because this was the cut-off for the original systematic review but also enough time had passed since the recommendation to see the effects of the implementation of NAATs in research studies. Further, we assessed for “small study effects” and publication bias with funnel plots followed by Egger’s test to determine their symmetry. We managed and analysed the data using Microsoft Excel 16 (Microsoft Corporation, USA) and R version 4.1.1 (R Foundation for Statistical Learning, Austria).

## Results

### Search results

**Fig. 2:**
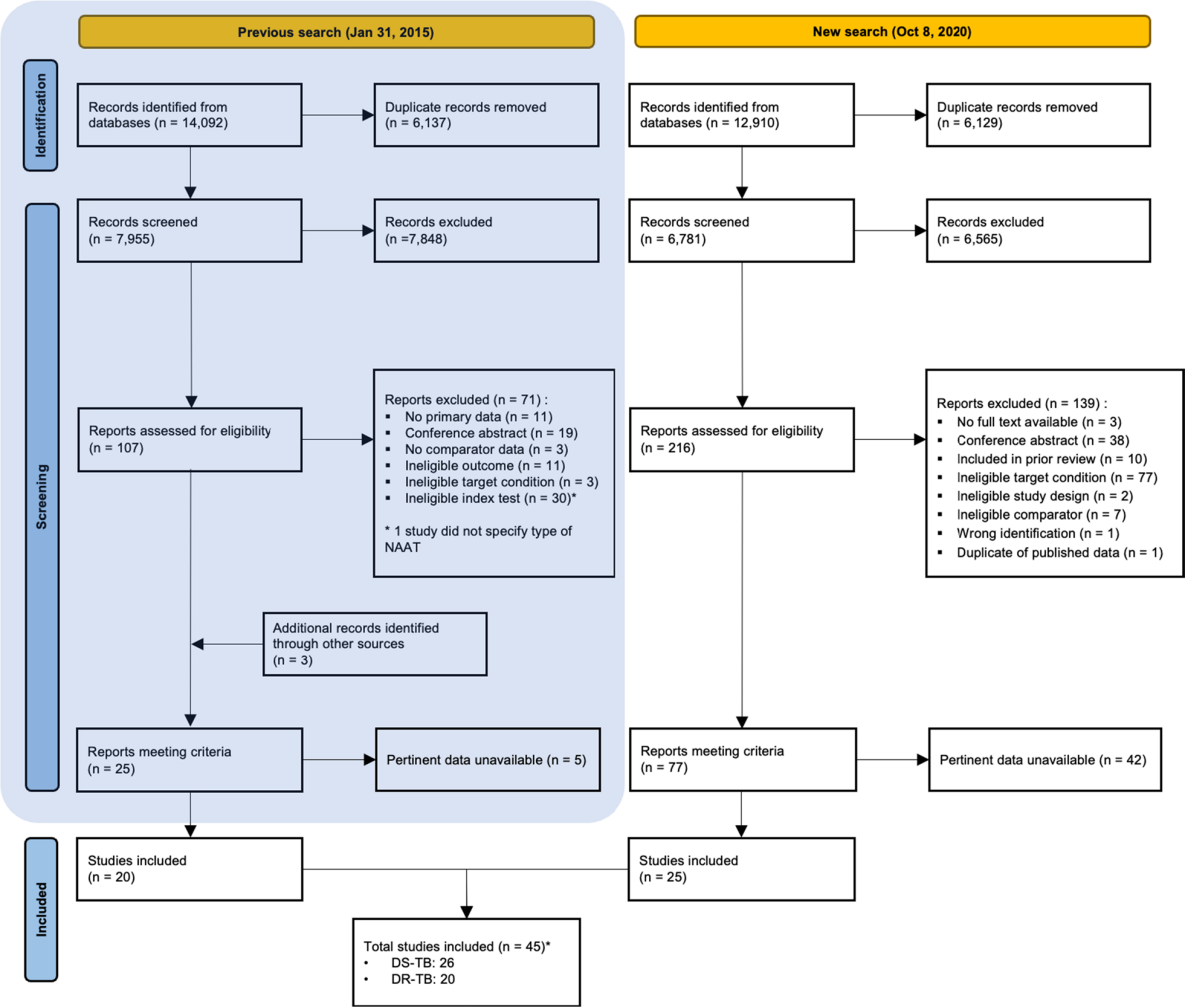
PRISMA diagram. *DS-TB* drug-sensitive tuberculosis, *DR-TB* drug-resistant tuberculosis. *One study [30] reported data for both DS-TB and DR-TB

After removing duplicates, we identified 14,776 (original review—7995; updated review—6781) titles and abstracts eligible for title and abstract screening. Of these, 323 were selected for full text review during screening. A total of 45 studies (26 DS-TB and 20 DR-TB) with relevant time delay estimates were ultimately included in this review (Fig. 2).

### Description of included studies

Of the 45 studies included in this review, 21 (81%) DS-TB and 15 (75%) DR-TB studies were conducted in Low-and Middle-Income Countries (LMICs) (Tables 1 and 2). One study had estimates for both DS-TB and DR-TB [30]. Overall, half of the studies (17 DS-TB, 7 DR-TB) were conducted in the African region with over two thirds of those in South Africa (n = 15). HIV prevalence was reported by 31 (19 DS-TB, 12 DR-TB) studies, of which about half (16 DS-TB, 4 DR-TB) reported a HIV prevalence of over than 50%. Amongst the DS-TB studies, 7 studies (27%) implemented Xpert as a point-of-care testing (POCT) program, and 15 studies (58%) implemented Xpert on-site, within walking distance of a primary care program or a laboratory.

**Table 1:** Study characteristics and time delays reported for diagnosis and treatment of drug-sensitive TB

Author	Year	Country	Study design	Setting	Level of healthcare system	HIV prevalence							
Boehme [30]	2011	Multiple^b^	Pre/post	Urban	Mixed	0.19							
Yoon [50]	2012	Uganda	Pre/post	Urban	Tertiary	0.76							
Kwak [51]	2013	South Korea	Observational	Urban	Tertiary	0.27							
Chaisson [52]	2014	USA	Hypothetical	Urban	Tertiary	NR							
Cohen [53]	2014	South Africa	Observational	Urban	Tertiary	1.00							
Cox [54]	2014	South Africa	Parallel Cl. RCT	Urban	Primary	0.60							
Durovni [32]	2014	Brazil	St.-we CI. RCT	Urban	Primary	0.10							
Mupfumi [34]	2014	Zimbabwe	Ind. RCT	Urban	Tertiary	1.00							
Sohn [55]	2014	Canada	Hypothetical	Urban	Tertiary	0.02							
Theron [37]	2014	Multiple^a^	Ind. RCT	Urban	Primary	0.6							
Calligaro [56]	2015	South Africa	Observational	Urban	Tertiary	0.27							
Muyoyeta [57]	2015	Zambia	Observational	Urban	Primary	0.52							
Page [35]	2015	Cambodia	Observational	NR	NR	NR							
Page [35]	2015	Kenya	Observational	NR	NR	NR							
Page [35]	2015	Swaziland	Observational	NR	NR	NR							
van den Handel [58]	2015	South Africa	Observational	Rural	Primary	0.28							
Hanrahan [59]	2016	Uganda	Observational	NR	NR	0.69							
Akanbi [60]	2017	Nigeria	Observational	Urban	Tertiary	1.00							
Calligaro [61]	2017	South Africa, Zimbabwe	Randomized, parallel group trial	Urban	Primary	0.58							
Mwansa-Kambafwile [62]	2017	South Africa	Observational	Urban	Primary	0.73							
Schmidt [63]	2017	South Africa	Observational	Rural	Primary	NR							
Shete [36]	2017	Uganda	Single arm interventional pilot	Rural	Primary	0.53							
de Castro [64]	2018	Brazil	Observational	Urban	Primary	0.05							
Khumsri [65]	2018	Thailand	RCT	Urban	Tertiary	NR							
Mugauri [66]	2018	Zimbabwe	Observational	Urban	Primary	NR							
Agizew [67]	2019	Bostwana	St.-we CI. RCT	NR	Primary	1							
Le [68]	2019	Vietnam	Observational	Rural	Tertiary	NR							
Nalugwa [69]	2020	Uganda	Observational	NR	Tertiary	0.838							

Author	Year	Diagnostic delay						Treatment initiation delay					
		Index		Comparator		Term	Time period	Index		Comparator		Term	Time period
		n	Median (IQR) (days)	n	Median (IQR) (days)			n	Median (IQR) (days)	n	Median (IQR) (days)		
Boehme [30]	2011	1429	1 day (0–2)	3659	Smear: 2 days (2–3) Culture: 58 days (42–62)	Time to detection	Collection of first sputum to receiving result by clinicians	1907	5 days^d^ (2–8)	4734	56 days (39–81)	Time to treatment initiation	First sputum collection to time to treatment initiation
Yoon [50]	2012	190	Same day (0–1)	246	1 day (0–26)	Time to detection	Enrolment to first positive result	190	6 days^d^ (1–61)	246	7 days (3–53)	Time-to-TB treatment	Enrolment to treatment initiation
Kwak [51]	2013	681	6 days (3–7)	681	Smear: 12 days (7–19.25) Culture: 38.5 (35.75–50.25)	Time to confirmation of receipt of results	Request of diagnostic test to confirmation of results by duty physician	43	7 days (4–9)	86	21 days (7–33.5)	Time to anti-TB treatment	Request of diagnostic test to initiation of ATT
Chaisson [52]	2014	142	1 day (0–2)	142	2 days (1–4)	Time to result	Order for admission to time to reporting results	NR	NR	NR	NR	NR	NR
Cohen [53]	2014	156	6.3 days (5.3–8.1)	90	3.3 days (2.1–5.2)	Total diagnostic time	Sputum collection to clinician receipt of result	NR	NR	NR	NR	NR	NR
Cox [54]	2014	NR	NR	NR	NR	NR	NR	982	4 days (2–8)	1003	8 days (2–27)	Time to TB treatment initiation	Enrolment^c^ to treatment initiation
Durovni [32]	2014	1385	7.3 days (3.4–9.0)	831	7.5 days (4.9–10.0)	Time to positive result	Specimen processing to lab-confirmed TB notification	1385	8.1 days (5.4–9.3)	831	11.4 days (8.5–14.5)	Time to treatment initiation	NR
Mupfumi [34]	2014	214	2 days (1–13)	210	6 days (1–25)	Time to diagnosis	Clinical presentation (baseline visit) to TB diagnosis	214	5 days (3–13)	210	8 days (3–23)	Time to treatment initiation	Clinical presentation (baseline visit) to treatment initiation
Sohn [55]	2014	11	1 day (0–4)	11	Smear: 1 day (1–2) Culture: 21.5 days (14–30)	Time to diagnosis	Time between first sample and the positive Xpert result	11	Hypothetically reduce by 12 days (4–23) in smear negative TB patient	11	26 days (4–30)	Time to reporting	Time from first sample to treatment initiation
Theron [37]	2014	NR	81% diagnosed on same day	NR	43% diagnosed on same day	NR	NR	744	Same day (0–3)	758	1 day (0–4)	Time to treatment	Enrolment^c^ to treatment initiation
Calligaro [56]	2015	111	0.2 days (0.2–0.3)	115	12.1 days (0.3–22.2)	Time to diagnosis	NA	111	0.3 days (0.2–1.2)	115	0.7 days (0.2–2.2)	Time to initiation of treatment	NR
Muyoyeta [57]	2015	NR	NR	NR	NR	NR	NR	553	2 days (1–5)	212	3 days (2–6)	Time to TB treatment	Date to first presentation to diagnostic services to date pt. was commenced on TB treatment
Page [35]	2015	NR	NR	NR	NR	NR	NR	15	16 days (6–33)	77	4 days (2–6)	Delay in treatment initiation	Collection of first specimen and treatment start
Page [35]	2015	NR	NR	NR	NR	NR	NR	17	4 days (2–7)	3	1 day (1–35)		
Page [35]	2015	NR	NR	NR	NR	NR		63	6.5 days (3–10)	7	8 days (1–11)		
van den Handel [58]	2015	NR	NR	NR	NR	NR	NR	75	1 day (0–2)	68	11.5 days (6–24)	Time to treatment	First sputum sample collection to anti-TB treatment initiation
Hanrahan [59]	2016	NR	NR	NR	NR	NR	NR	48	0 days (0–0)	39	Empiric: 14 days (5–35) Culture: 144 days (28–180)	Time to treatment	Sputum collection to TB treatment
Akanbi [60]	2017	NR	NR	NR	NR	NR	NR	56	5 days (2–8)	20	12 days (5–35)	Time to treatment	Baseline visit (specimen collection) to treatment initiation
Calligaro [61]	2017	NR	NR	NR	NR	NR	NR	435	1 day (0.1–4)	413	4 days (1–31)	Time-to-treatment initiation	Enrolment to initiated on treatment
Mwansa-Kambafwile [62]	2017	NR	NR	NR	NR	NR	NR	177	0 days (0–0)	21	9 days (4–20)	Time to treatment initiation	NR to treatment initiation
Schmidt [63]	2017	851	0 days (0–1)	738	2 days (1–22)	Time to laboratory diagnosis	Sputum sample collection to test result reported	851	4 days (2–8)	NR	NR	Time to TB treatment initiation	Time from sputum sample collection to time when TB treatment was recorded as being initiated
Shete [36]	2017	1091	1 day (0–2)	54	1 day (0–2)	Time-to-diagnosis	NR	41	6 days (2–11)	113	1 day (0–1)	Time-to-treatment	NR
de Castro [64]	2018	24	6 days (2–8)	41	3 days (2–6)	Time from triage to NR	Triage to lab test result release	24	14.5 days (8–28)	41	8 days (6–12)	Time from triage to NR	Triage to treatment initiation
Khumsri [65]	2018	40	1.88^e^ (SD 1.07)	36	4.11^e^ (SD 2.22)	Time to get correct diagnosis	Outpatient department visit to receive correct diagnosis	NR	NR	NR	NR	NR	NR
Mugauri [66]	2018	NR	NR	NR	NR	NR	NR	340	20.17 days^e^ (SD 10.3)	318	22.44 days^e^ (SD 30.2)	Delay in treatment initiation from diagnosis	Diagnosis to treatment initiation
Agizew [67]	2019	NR	NR	NR	NR	NR	NR	159	6 days (2–17)	42	22 days (3–51)	Time-to-treatment	Sputum collection to treatment initiation
Le [68]	2019	69	2 days (1–4)	NR	NR	NR	NR	69	1 day (0–1)	8	3 days (1–8)	Time to anti-TB treatment	Hospital admission to treatment initiation
Nalugwa [69]	2020	NR	NR	NR	NR	NR	NR	33	2 days (0–14)	NR	0 days (0–1)	Time to treatment	NR to treatment initiation

Page 2015 reported time delay for 4 different sites, 3 of which were included in the analysis as individual studies; the one remaining site did not have time delay data on smear and was excluded from the primary analysis

van den Handel 2015 compared Xpert in both centralized and decentralized settings with smear, and they were also separately included in the analysis

POCT programs were generally defined by each study as performing Xpert testing by non-laboratory personnel within the TB clinic

Countries were classified using the World Bank classification based on gross national income (GNI) in 2015 for studies that were included in the original search and 2020 for studies included in the updated search

Ind. RCT: Individually Randomized Controlled Trial; Cl. RCT: Cluster Randomized Controlled Trial; St.-we. Cl. RCT: stepped-wedge Cluster Randomized Controlled Trial; pre/post: pre/post implementation study; hypothetical: single-cohort hypothetical study; observational: single-cohort observational study; POCT: point-of-care testing; TB: tuberculosis; MDR: multidrug-resistant tuberculosis; NR: not reported

^a^South Africa, Peru, India, Azerbaijan, Philippines, and Uganda

^b^South Africa, Zimbabwe, Zambia, and Tanzania

^c^Estimated based on study design

^d^In smear negatives

^e^Reported means and standard deviations

**Table 2:** Study characteristics and time delays reported for diagnosis and treatment of drug-resistant TB

Author	Year	Country	Study design	Setting	Level of healthcare system	HIV prevalence	Type of culture	Type of LPA					
Boehme [30]	2011	Multiple^a^	Observational	Urban	Mixed	0.19	Both	Both					
Chryssanthou [70]	2011	Sweden	Observational	Urban	Tertiary	NR	Liquid culture	Direct					
Skenders [71]	2011	Latvia	Observational	NR	NR	NR	Liquid culture	Direct					
Hanrahan [40]	2012	South Africa	Observational	NR	NR	0.58	Liquid culture	Both					
Jacobson [72]	2012	South Africa	Observational	Rural	Tertiary	0.30	Liquid culture	Indirect					
Lyu [73]	2013	South Korea	Observational	Urban	Tertiary	0.01	Liquid culture	Direct					
Gauthier [74]	2014	Haiti	Observational	Urban	Tertiary	NR	Solid culture	Direct					
							Liquid culture						
Kipiani [75]	2014	Georgia	Observational	Urban	Tertiary	0.03	Solid culture	Direct					
Raizada [76]	2014	India	Observational	Urban	NR	NR	Solid culture	Direct					
Singla [77]	2014	India	Observational	Urban	Tertiary	NR	Both	Direct					
Bablishvili [78]	2015	Georgia	Observational	Urban	Tertiary	NR	Solid culture	Direct					
							Liquid culture						
Cox [39]	2015	South Africa	Observational	Urban	Primary	0.74	Both	Direct					
Eliseev^b^ [79]	2016	Russia	Observational	NR	NR	0.06	Solid culture	Direct					
							Liquid culture						
Evans [80]	2017	South Africa	Observational	Urban	Tertiary	0.89	NR	NR					
Iruedo [81]	2017	South Africa	Observational	Rural	Primary	0.61	NR	NR					
Evans [82]	2018	South Africa	Observational	Urban	Primary	0.26	NR	NR					
Li^b,c^ [43]	2019	China	Observational	Urban	Tertiary	NR	Solid culture	Direct					
Jeon [41]	2020	South Korea	Observational	Urban	Tertiary	NR	NR	NR					
Ngabonziza [83]	2020	Rwanda	Observational	NR	Tertiary	0.40	NR	NR					
Shi [42]	2020	China	Observational	Urban	Tertiary	NR	Solid culture	Direct					

Author	Year	Diagnostic delay						Treatment initiation delay					
		Index		Comparator		Term	Time period	Index		Comparator		Term	Time period
		n	Median (IQR) (days)	n	Median (IQR) (days)			n	Median (IQR) (days)	n	Median (IQR) (days)		
Boehme [30]	2011	244	63 (38–102)	356	40 (27–53)	Time to detection	Specimen collection to receiving result by clinicians	NR	NR	NR	NR	NR	NR
Chryssanthou [70]	2011	127	21 (13–78)	127	7 (1–16)	Lab processing time	Specimen arrival at lab to report of DST to clinician	NR	NR	NR	NR	NR	NR
Skenders [71]	2011		NR	NR	NR	NR	NR	47	40 (23–67)	22	14 (7–22)	Admission to treatment start	Hospital admission to treatment start
Hanrahan [40]	2012	1176	52 (41–77)	1177	26 (11–52)	Test turnaround time	Date of sputum collection to DST results	26	78 (52–93)	52	62 (32–86)	Time to MDR-TB treatment	Date of sputum collection to MDR-TB treatment
Jacobson [72]	2012	89	55 (46–66)	108	27 (20–34)	Lab processing time	Specimen arrival at lab to report of results	89	80 (62–100)	108	55 (37.5–78)	Time to MDR treatment initiation	Specimen collection to MDR treatment initiation
Lyu [73]	2013	428	83 (68–92)	168	12.7 (8–17)	Turnaround time	Test request to reporting of results	NR	NR	NR	NR	NR	NR
Gauthier [74]	2014	221	54 (43–64)	221	7.5 (6.5–8.5)	Turnaround time	NR to time to positivity	NR	NR	NR	NR	NR	NR
		221	19 (12–25)	221	7.5 (6.5–8.5)	Turnaround time	NR to time to positivity	NR	NR	NR	NR	NR	NR
Kipiani [75]	2014	NR	NR	NR	NR	NR		72	83.9 (56–106)	80	18.2 (11–24)	Time to MDR-TB treatment initiation	Sputum collection to start of SLD therapy
Raizada [76]	2014	248	87 (42–208)	248	11 (1–76)	Turnaround testing time	Specimen collection to DST result being available	NR	NR	NR	NR	NR	NR
Singla [77]	2014	121	107 (79–131)	433	5 (3–6)	Diagnostic time in lab	Specimen arrival at lab to MDR-TB report	51	157 (127–200)	83	38 (30–79)	NR	Time from identification patients suspected for MDR-TB to MDR-TB treatment initiation
Bablishvili [78]	2015	155	33 (27–41)	336	5 (3–7)	Time to MTB detection	Sample collection to recorded results	NR	NR	NR	NR	NR	NR
		227	9 (7–11)	336	5 (3–7)	Time to MTB detection	Sample collection to recorded results	NR	NR	NR	NR	NR	NR
Cox [39]	2015	NR	NR	NR	NR	NR	NR	95	76 (62–111)	173	28 (16–40)	Time to treatment	Time from collection to treatment initiation
Eliseev^b^ [79]	2016	NR	NR	NR	NR	NR	NR	38	90 (76.3–117.3)	72	24 (19–51)	Time to MDR-TB treatment initiation	First visit to treatment
		NR	NR	NR	NR	NR	NR	58	74 (55–99.8)	72	24 (19–51)	Time to MDR-TB treatment initiation	First visit to treatment
Evans [80]	2017	NR	NR	NR	NR	NR	NR	256	81 (49–115)	256	38 (23–54)	NR	Sputum collection to treatment initiation
Iruedo [81]	2017	143	45 (39–59)	28	11.5 (8–21)	Time to diagnosis	Sputum collection to issue of report to clinic	143	64 (50–103)	28	29 (14.5–53)	Time to treatment	Sputum collection to treatment initiation
Evans [82]	2018	NR	NR	NR	NR	NR	NR	7	81 (28–97)	129	38 (23–51)	Time to treatment initiation	Specimen collection to treatment initiation
Li^b,c^ [43]	2019	155	53 (49–60)	155	3 (2–4)	Turnaround time	Sample receipt to reporting date of results	NR	NR	NR	NR	NR	NR
Jeon [41]	2020	NR	NR	NR	NR	NR	NR	263	13 (5–25)	202	5 (2–9.3)	Time to MDR treatment initiation	MDR-TB diagnosis to MDR-TB treatment initiation
Ngabonziza [83]	2020	313	87 (78–98)	197	40 (25–55)	RR-TB diagnostic delay	Specimen collection to results being available	NR	NR	NR	NR	NR	NR
Shi [42]	2020	105	62 (53–69)	113	16 (10–19)	NR	NR	37	69 (59–77)	42	19 (14–23)	NR	NR

Evans 2017 compares different diagnostic methods within the cohort, whereas Evans 2018 compares cohort analysed in Evans 2017 with a later cohort

Countries were classified using the World Bank classification based on gross national income (GNI) in 2015 for studies that were included in the original search and 2020 for studies included in the updated search

*LPA* line probe assay, *IQR* interquartile range, *NR* not reported, *DST* drug susceptibility testing, *MDR* multidrug resistance, *SLD* second line drug therapy, *RR-TB* rifampin-resistant TB, *MTB* mycobacterium tuberculosis

^a^South Africa, Zimbabwe, Zambia, and Tanzania

^b^Estimates only for sputum smear positive patients

^c^Reported means and standard deviations

### Quality assessment of time delay estimates

The studies had considerable methodological heterogeneity in the definitions of time delays. When classifying reported time delays according to our operational definitions and by study design, no study reported all sub-components of time delay. All studies evaluating treatment delay used TB treatment initiation time but start and end points for diagnostic delay varied across studies (Tables 1 and 2). Overall, 13 of the 45 studies did not provide a clear definition of the time delay estimates reported (Table 3). Amongst studies included in the DS-TB analysis, 6 (23%) studies employed a randomized control trial (RCT), and 2 studies (8%) were quasi-experimental using pre- and post-implementation study designs. One study used a single-arm interventional pilot study (4%), and the remaining 15 studies were observational (58%). All the studies in the DR-TB analysis were observational. In the use of proper statistical methods for measurement and reporting of delay estimates, 18 studies ranked high, 23 ranked medium, and 2 ranked low. In the evaluation of time estimates alongside patient important outcomes, 7 ranked high, 18 ranked medium, and 18 ranked low.

**Table 3:**
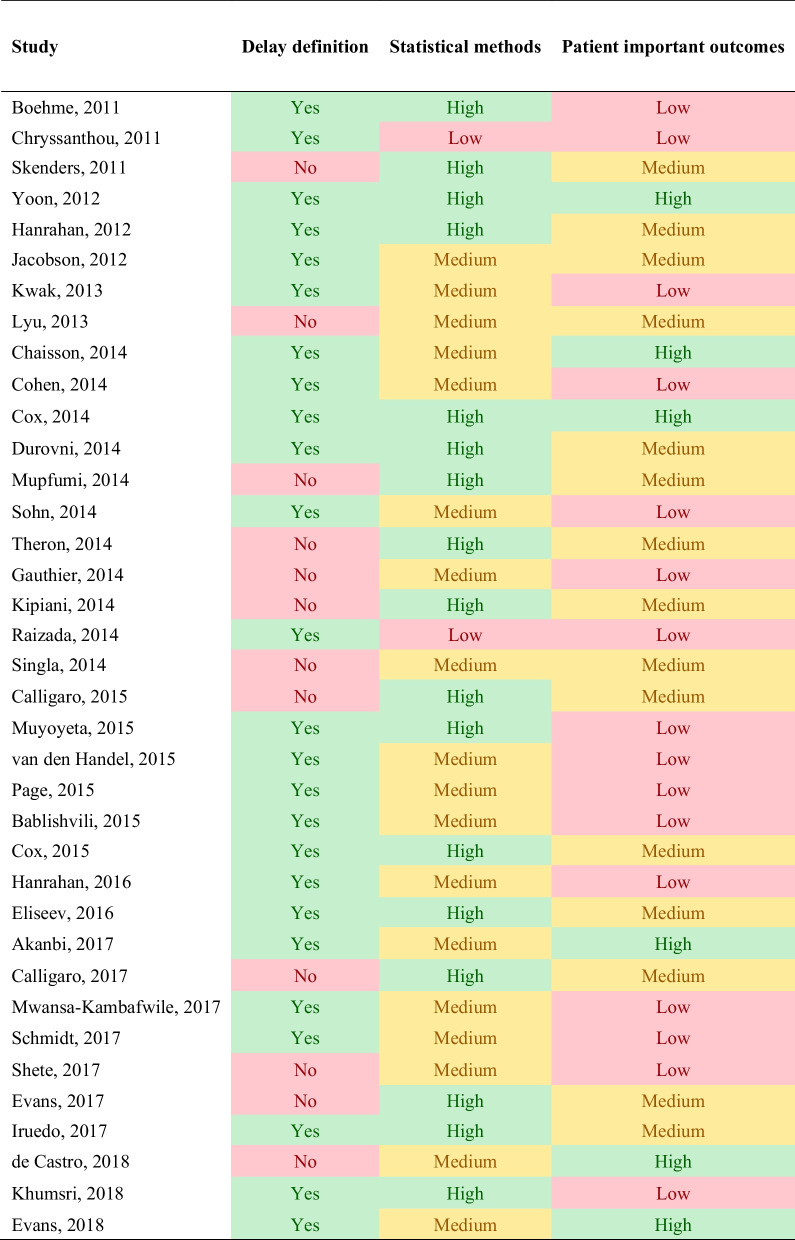

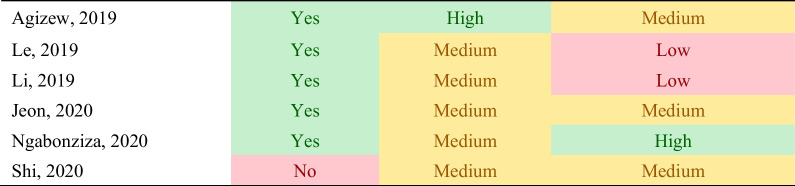
Quality assessment of time delay estimates

1. Delay definition: provision of clear definition of measuring time delay and reporting the time delay estimates

2. Statistical methods: use of appropriate statistical methods to report and assess changes in time delays

3. Patient important outcomes: evaluating time estimates alongside patient-important outcomes

The color shades in red, yellow, green indicate study quality from low to high within each category

In all funnel plots (Additional file 2), there were several studies falling outside of the 95% CI, impacting the visualized asymmetry. This may be due to considerable heterogeneity (I^2^ > 99%) of the studies. However, Egger’s tests—used to assess whether there are systematic differences between high- and low-precision studies—demonstrated no clear evidence of “small study effects.” (p = 0.085–0.462).

### Impact of NAATs on delay

**Fig. 3:**
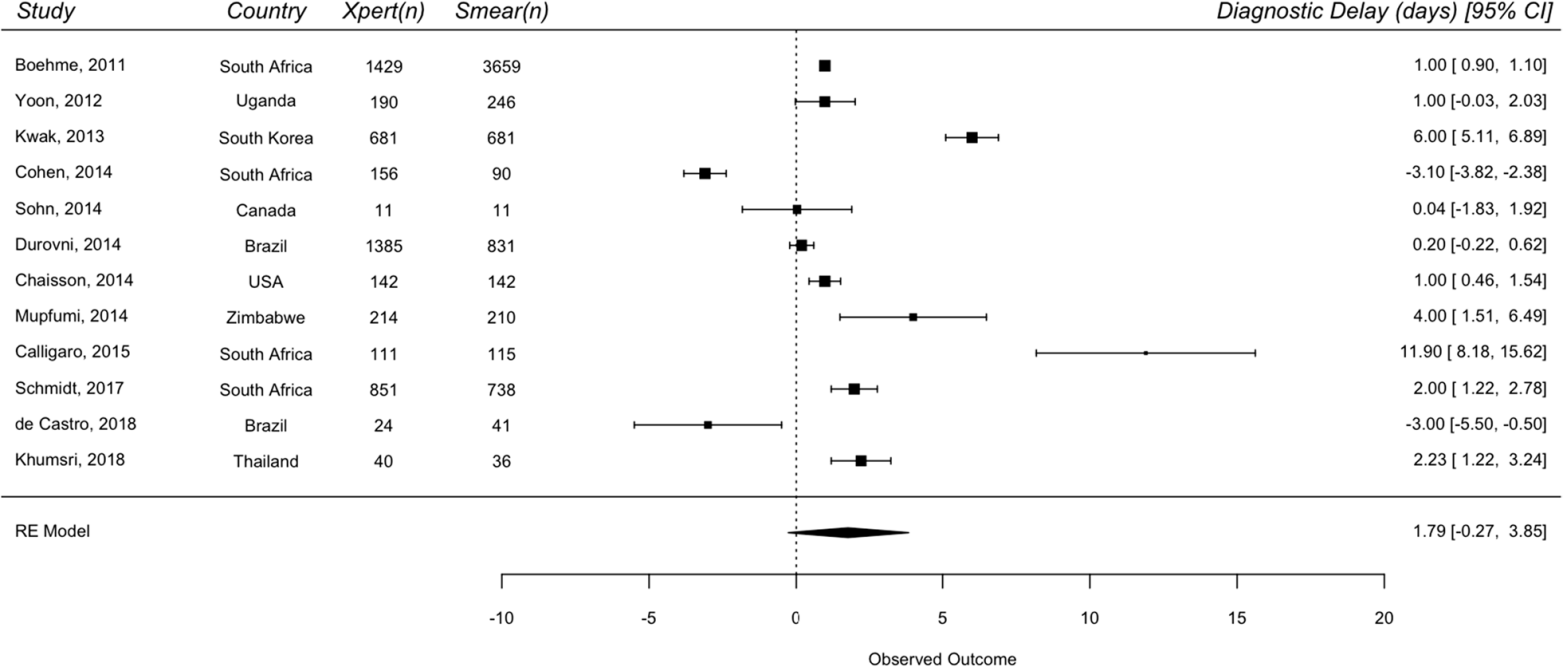
Forest plots of raw median difference in diagnostic delay for Xpert and smear for drug-sensitive TB

**Fig. 4:**
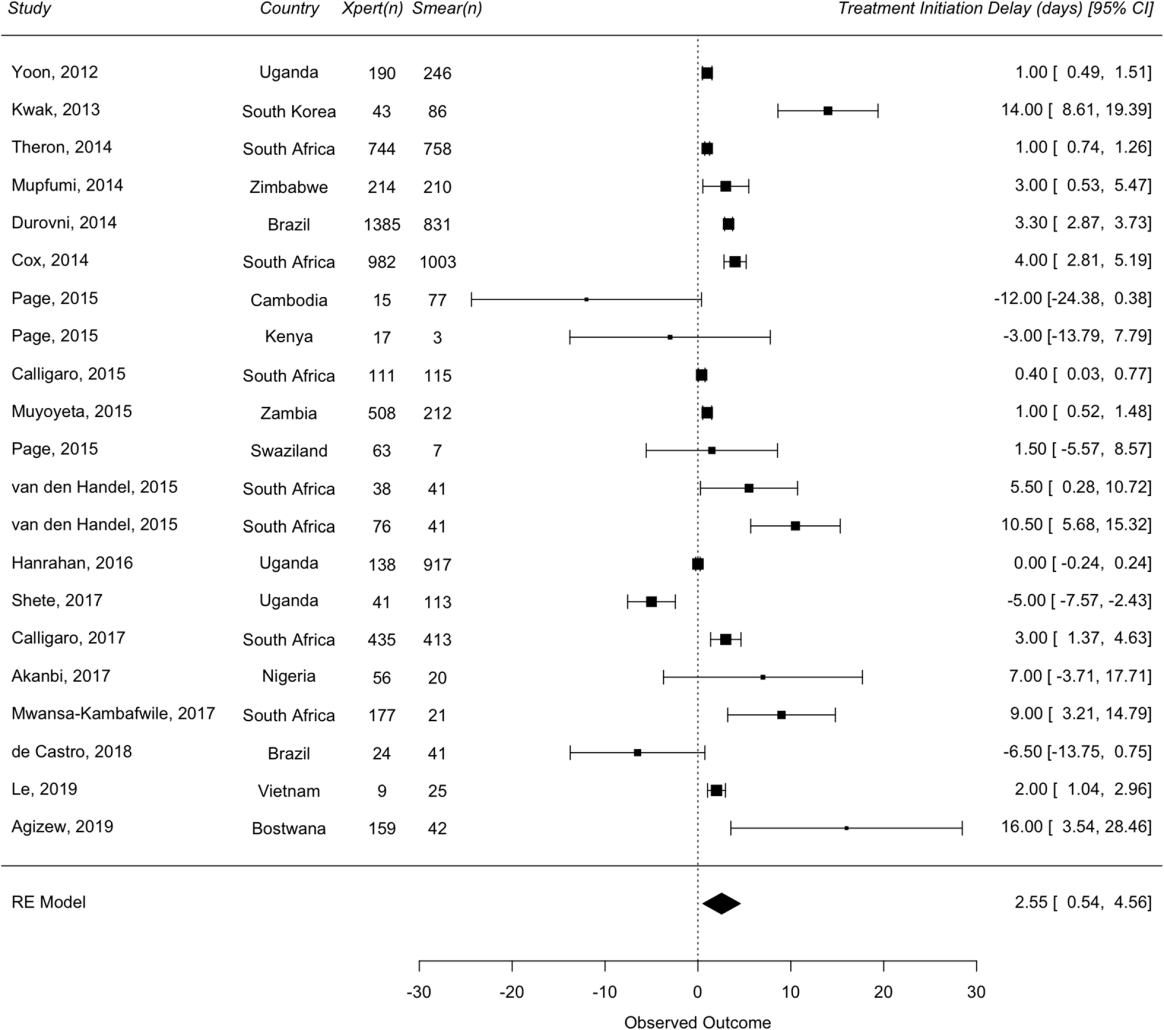
Forest plots of raw median difference in treatment initiation delay for Xpert and smear for drug-sensitive TB

For DS-TB analysis, 12 studies were included in the primary analysis for diagnostic delay, and 18 studies were included for treatment initiation delay. The overall median diagnostic delay for smear and Xpert were 3 days and 1.04 days, respectively. The overall median treatment initiation delay for smear and Xpert were 6 days and 4.5 days, respectively. A random effects meta-analysis of the difference of medians showed that the use of Xpert did not show a statistically significant reduction in diagnostic delay [1.79 days (95% CI − 0.27 to 3.85)] compared to smear but showed a statistically significant reduction in treatment initiation delay by 2.55 days (95% CI 0.54–4.56) (Figs. 3 and 4).

**Fig. 5:**
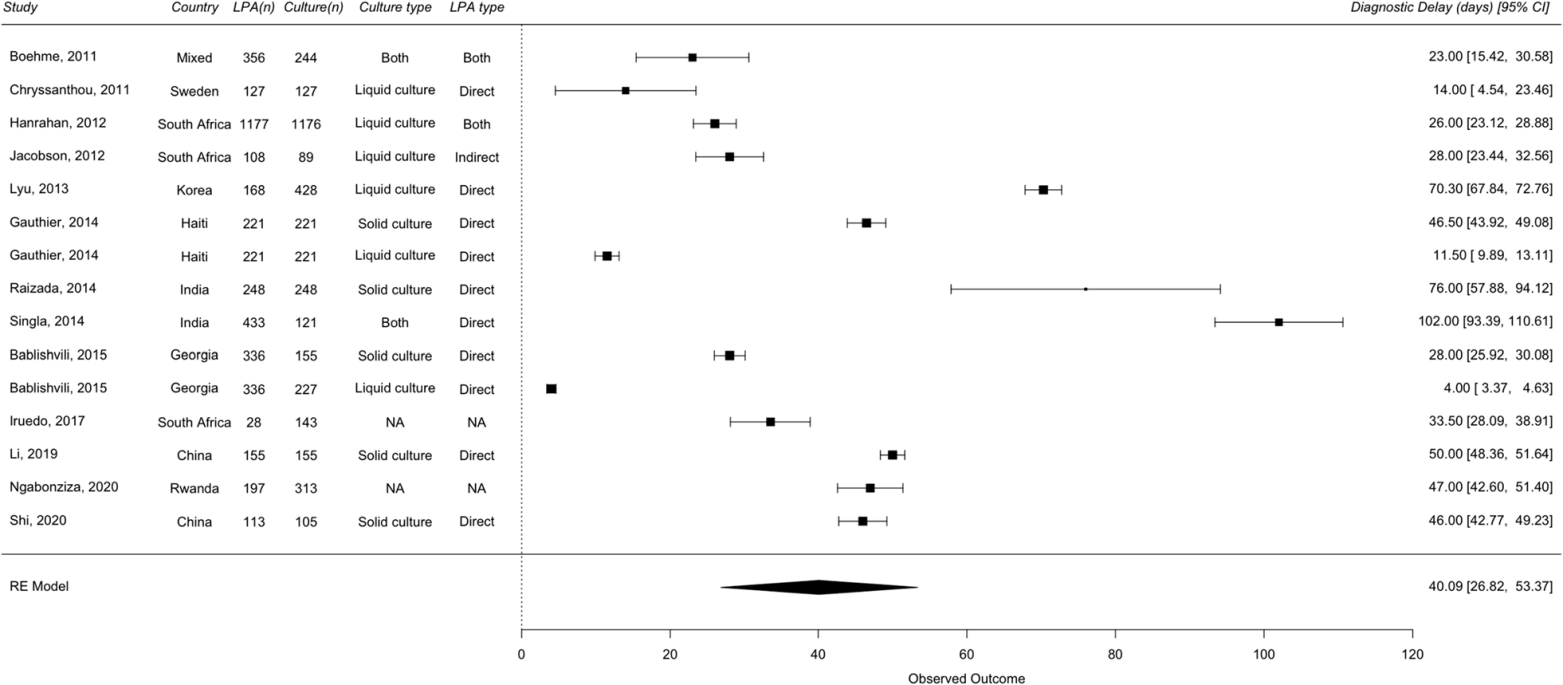
Forest plots of raw median difference in diagnostic delay for LPA and culture DST for drug-resistant TB

**Fig. 6:**
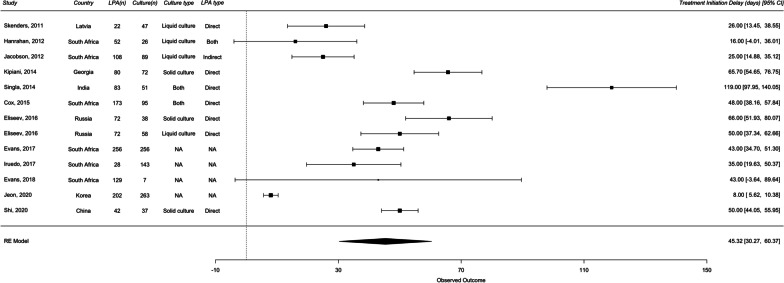
Forest plots of raw median difference in treatment initiation delay LPA and culture DST for drug-resistant TB

For DR-TB analysis, 13 studies were included in diagnostic delays and 12 studies were included in treatment initiation delays. The overall median diagnostic delay for culture DST and LPA were 54 days and 11 days, respectively. The overall median treatment initiation delay for culture DST and LPA were 78 days and 28 days, respectively. A random effects meta-analysis of the difference of medians showed that, in comparison with culture DST, the use of LPA significantly reduced diagnostic delay by 40.09 days (95% CI 26.82–53.37) and treatment initiation delay by 45.32 days (95% CI 30.27–60.37) (Figs. 5 and 6). I^2^ value of 99.79% and 97.22% for diagnostic and treatment initiation delay indicated considerable heterogeneity.

Comparing the studies from the two different phases of the review (pre-/post-2015), we found no statistical significance in the reduction of diagnostic delays but observed statistical significance in the reduction of treatment initiation delay with a median difference of 2.54 days (95% CI 0.45–4.62) for post-2015 studies and 5.04 days (95% CI 0.09–9.99) for pre-2015 studies. Similarly, subgroup analysis based on study design showed a statistically significant reduction in treatment initiation delay in the RCT group [2.85 days (95% CI 1.16–4.55)] but not in the observational group [1.67 days (95% CI − 1.70 to 5.05)]. When classifying studies by the healthcare systems level, Xpert did not provide meaningful reduction in treatment initiation delay regardless of the location of its placement: 1.27 days (95% CI − 1.45 to 4.00) for primary health care centres and 5.27 days (95% CI − 1.06 to 11.60) for tertiary hospitals. When grouped by POCT status, Xpert test implemented as a POCT service showed statistically significant reductions in treatment initiation delay compared to non-POCT programs. All sub-group analyses with greater than 2 studies showed I^2^ values greater than 89%, suggesting considerable heterogeneity (Tables 4 and 5).

**Table 4:** Subgroup analyses of reported time delay for TB diagnosis

Subgroup	# of studies	Median reduction (95% CI)	I^2^	p-value
**Year**				
Pre-2015	8	1.21 (− 0.67 to 3.10)	98.99%	0.20
Post-2015	4	3.15 (− 2.72 to 9.01)	98.56%	0.29
**Empiric treatment rate**				
High ≥ 50%	5	1.58 (0.55–2.61)	89.18%	0.003
Low < 50%	7	1.85 (− 1.91 to 5.6)	99.31%	0.34
**HIV prevalence**				
High ≥ 50%	5	1.12 (− 1.19 to 3.43)	96.31%	0.34
Low < 50%	7	2.31 (− 1.09 to 5.71)	99.62%	0.18
**Healthcare level**				
Primary	4	0.83 (− 1.83 to 3.49)	96.25%	0.54
Tertiary	7	2.54 (− 0.84 to 5.92)	98.87%	0.14
**Study design**				
RCT	3	4.56 (− 2.72 to 11.43)	99.19%	0.19
Observational	6	1.17 (− 1.18 to 4.1)	97.85%	0.44
**Overall**	12	1.78 (− 0.27 to 3.85)	99.25%	0.089

Pre-2015 refer to studies with data from before 2015 when Xpert capacity was limited. For empiric treatment rate and HIV prevalence, 50% or greater was considered to be high

*RCT* Randomized Controlled Trial

**Table 5:** Subgroup analyses of reported time delay for TB treatment

Subgroup	# of studies	Median reduction (95% CI)	I^2^	p-value
**Year**				
Pre-2015	6	5.04 (0.09–9.99)	99.64%	0.046
Post-2015	12	2.54 (0.45–4.62)	98.86%	0.017
**Empiric treatment rate**				
High ≥ 50%	12	2.64 (0.93–4.35)	98.34%	0.002
Low < 50%	6	1.71 (− 4.24 to 7.66)	98.81%	0.56
**HIV prevalence**				
High ≥ 50%	12	1.09 (− 0.78 to 2.95)	98.36%	0.25
Low < 50%	6	4.61 (− 0.79 to 10.00)	99.50%	0.09
**Healthcare level**				
Primary	8	1.27 (− 1.45 to 4.00)	99.07%	0.36
Tertiary	4	5.27 (− 1.06 to 11.60)	98.87%	0.1
**POCT program**				
POCT	7	3.98 (1.13–6.81)	99.23%	0.0061
Lab	11	0.79 (− 2.75 to 4.33)	99.27%	0.66
**Study design**				
RCT	5	2.85 (1.16–4.55)	95.44%	0.001
Observational	13	1.67 (− 1.70 to 5.05)	99.57%	0.33
**Overall**	21	2.55 (0.54–4.56)	99.31%	0.013

Pre-2015 refer to studies with data from before 2015 when Xpert capacity was limited. For empiric treatment rate and HIV prevalence, 50% or greater was considered to be high

*POCT* point-of-care testing, *RCT* Randomized Controlled Trial

## Discussion

### Principal findings

While there are several patient-important impact measures for new diagnostic tests [31], time delay estimates provide direct measure of the timeliness of TB care. To our knowledge, our systematic review of 45 studies is the first to comparatively synthesize and quantify reductions in delays in diagnosis and treatment of DS and DR-TB when the WHO recommended NAATs are used instead of smear (DS-TB) or culture DST (DR-TB). Our random effectives meta-analysis of the differences of median times showed that the use of NAATs improved treatment initiation delay for patients investigated for both DS and DR-TB; however, this benefit was not seen for diagnostic delay for DS-TB (Xpert vs. smear). We also found that the degree of benefit in reducing delays in using NAATs for TB care was highly variable and dependent on how the tests were implemented (e.g., laboratory-based vs. POCT), differences in study design to evaluate impact of NAATs on TB care delays, and large variations in how delays were defined and quantified.

In principle, Xpert and smear are “same-day” tests; therefore, expected reduction in diagnostic delays may be limited for Xpert. As such, in our meta-analysis, we did not find significant reduction in diagnostic delays when using Xpert compared to smear [1.79 days (95% CI − 0.27 to 3.85)]. For treatment delays, our analysis of 18 studies showed that Xpert reduced treatment initiation delays for DS-TB by 2.55 days (95% CI 0.54–4.56) compared to smear, but the degree of this effect was highly variable depending on how and where Xpert was deployed within the health care system. Particularly, in our sub-group analysis, we found that the use of Xpert as non-POCT (at any levels of health system) did not show meaningful improvement in DS-TB treatment initiation delay. Moreover, the ‘hub-and-spokes’ model—where patient samples for Xpert from several community health centres (spokes) are referred to a centralized laboratory (hub) in the system—for Xpert testing evaluated in earlier studies has shown limited impact on improving and optimizing the timeliness of TB care due to operational barriers causing further delays [32–34], de-prioritization of Xpert use as an initial test in the national algorithms [35, 36], and continued high empiric treatment [37, 38] rates in certain settings.

In contrast to DS-TB, use of LPA for DR-TB care had resulted in large reduction in delays for DR-TB care. Our meta-analysis results found that use of LPA drastically reduced overall DR-TB care delays by 45.32 days (95% CI 30.27–60.37). This was mainly due to prolonged delays associated with conventional DR-TB diagnostics (culture DST) that takes weeks to diagnose and treat DR-TB patients. However, reduction of these delays were not solely due the implementation of the technology alone. In an earlier phases of LPA implementation in South Africa, use of LPA for DR-TB care were much restricted and centralized at higher levels of the health and laboratory system, and caused treatment initiation delays of more than 50 days [39, 40]. DR-TB care delays gradually improved to 28 days (IQR: 16–40) through the 3-year DR-TB care decentralization program, which included streamlining LPA testing in the clinical practice (years 2009–2011). Moreover, studies from settings with more established healthcare infrastructure (e.g., China and South Korea) also found that operational challenges diminished the potential benefit of rapid molecular testing in improving DR-TB care delays [41–43].

### Strengths and limitations

For the meta-analysis, we used the Quantile Estimation (QE) method because it had excellent performance in simulation studies that were motivated by our systematic review [26]. One advantage compared to more traditional approaches based on meta-analysing the difference of means is that the QE method uses an effect size that is typically reported by the primary studies (i.e., the difference of medians) rather than one that must be estimated from the summary data of the primary studies (i.e., the difference of means). However, our meta-analysis results should be interpreted with caution because considerable statistical power was lost when restricting to studies that presented all the necessary data for estimating the variance of the difference of medians. Also, the high level of clinical (e.g. participants, outcomes) and methodological heterogeneity (e.g. study design, defining and reporting of time delays) in the studies included in our review translated into high I^2^ values in all of our meta-analyses results, making generalized interpretation of our summary estimates difficult. We also advise caution in the interpretation of our subgroup analyses because these confounders often complicate the interpretation and lead to wrong conclusions [44].

Delays in TB care occur due to a wide range of patient and health systems risk factors. [46, 48] Studies included in our review did not comparatively assess and adjust for risk factors associated with time delays for both the index (Xpert or LPA) and the comparator (smear or culture DST). This may be because time delay estimates were not the primary outcomes in most of the studies, and thus lacking proper analytical assessment of these outcome measures. Therefore, we were limited to sub-group analyses on key study-level attributes (e.g., HIV prevalence, empiric treatment rate, Xpert placement strategy, and study design), which were highly heterogenous and in many cases, inconclusive in showing that Xpert improved delays in TB care. Moreover, our findings are subject to potential confounding issues—at both health systems (e.g., differences in healthcare system infrastructure, TB care practices, implementation strategies of the index tests) and patient level factors (e.g., symptom levels, age, care-seeking behaviours)—which may bias our effect estimates (number of days reduced in diagnostic and treatment initiation delays) towards or away from the null. Given these reasons, generalizability of our findings may be limited. Likewise, our review underscores a need for more research investigating health systems and patient factors that can impact delays in TB care during and after the implementation of diagnostic tests and strategies that aim to improve the timeliness and quality of TB care. Lastly, despite carrying out comprehensive searches and considering non-English studies, we may have missed some studies in our review. Therefore, we cannot rule out potential publication bias.

In our study, we also investigated consistencies in defining and reporting of time delays across studies with a framework developed as part of our study (Fig. 1). In our quality assessment of the studies reporting time delay estimates (Table 3), we found considerable heterogeneity in defining time delays and close to 30% of studies (13) reported delay estimates without providing clear definitions. Many of the studies included in our review used the same terms to define different components of the delay. For instance, “turnaround time”, “time to detection”, and “laboratory processing time” were used to describe the time from specimen receipt by the lab to test result at the lab, while others employed these same terms to define diagnostic delay, time from specimen collection to notifying the clinic of the test result. In addition, several studies included in our review did not include or inappropriately reported uncertainty ranges (e.g., no IQRs or reported means with IQRs). As time data may be highly skewed, standardizing the practice of reporting delay estimates as medians with their variances or other measures of spread (e.g., IQR or range) can help facilitate synthesis of these studies. Many of these issues have been previously reported by other systematic reviews on TB care delays and our findings reemphasizes the importance in standardizing how TB care delays are defined, measured, and reported [20, 45–48].

## Conclusions

The global rollout of NAATs has dramatically changed the landscape of TB diagnosis in high TB burden settings with improvements in the TB diagnostic infrastructure and the quality of TB prevention and care programs. Our systematic review findings suggest that implementation of NAATs have resulted in a noticeable reduction in delays for TB treatment compared to the conventional methods. However, these improvements did not fully realize the potential benefits of NAATs because of health system limitations [49]. Additionally, we identified methodological concerns in reporting of time delay estimates and emphasize the need to standardize and promote their consistent reporting.

## Supplementary Information

**Additional file 1.** Systematic review search strategy. The detailed search strategy for each database searched for this review.

**Additional file 2.** Funnel plots. Outputs from the analysis of risk of bias.

**Additional file 3.** Data extraction tool. The template for the data extraction tool.

## Data Availability

All data generated or analysed during this systematic review are included in this published article and the additional files.

## Abbreviations

DS-TB: Drug-sensitive tuberculosis
DR-TB: Drug-resistant tuberculosis
LPA: Line probe assay
NAAT: Nucleic acid amplification test
POCT: Point-of-care test
QE: Quantile estimation
TB: Tuberculosis
WHO: World Health Organization
