# Supplementary material for: Impact of molecular diagnostic tests on diagnostic and treatment delays in tuberculosis: a systematic review and meta-analysis
Source: BMC Infect Dis. 2022 Dec 14;22:940. doi: 10.1186/s12879-022-07855-9 (PMC9748908; doi:10.1186/s12879-022-07855-9)
Supplement: Supplementary file 1 — Additional file 1. Systematic review search strategy. The detailed search strategy for each database searched for this review. [file 12879_2022_7855_MOESM1_ESM.docx]

**TB-NAATs-PCOs: Revised Search Strategies for Search Update**

**Date run: 12October2020; Date limit applied: from January 1, 2015**

- **Ovid Medline 3,214 results**
- **Embase.com 4,846 results**
- **Web of Science 3,208 results**
- **Global Health 1,642 results**

**Database: Medline**

1. exp Tuberculosis/

2. Mycobacterium tuberculosis/

3. tuberculosis.mp.

4. tb.tw.

5. or/1-4

6. Nucleic Acid Amplification Techniques/

7. Molecular Diagnostic Techniques/

8. nucleic acid test*.tw.

9. NAAT.tw.

10. NAATs.tw.

11. NAA.tw.

12. direct amplification.tw.

13. transcription-mediated amplification.tw.

14. RNA amplification*.tw.

15. DNA amplification*.tw.

16. molecular assay*.tw.

17. molecular diagnos*.tw.

18. polymerase chain reaction*.tw.

19. PCR.tw.

20. PCRs.tw.

21. Xpert.tw.

22. GeneXpert.tw.

23. cepheid.tw.

24. "MTB/RIF".tw.

25. cobas.tw.

26. TaqMan.tw.

27. AMTD*.tw.

28. MTD.tw.

29. Gen-Probe.tw.

30. ligase chain reaction*.tw.

31. LCx.tw.

32. line probe assay*.tw.

33. LPA.tw.

34. LPAs.tw.

35. AMTD*.tw.

36. MTBDR*.tw.

37. gMTBDR.tw.

38. INNO-LiPA.tw.

39. ProbeTec.tw.

40. loopamp.tw.

41. EXPAR.tw.

42. LAMP.tw.

43. loop mediated amplification*.tw.

44. Exponential Amplification Reaction*.tw.

45. NALF.tw.

46. nucleic acid lateral flow*.tw.

47. (nucleic acid and amplification).tw.

48. (NAT or NATs).ti.

49. (amplified and direct test*).tw.

50. BD Probe.tw.

51. Tec Direct.tw.

52. or/6-51

53. exp Time/

54. Delayed Diagnosis/

55. Early Diagnosis/

56. Comparative Effectiveness Research/

57. Follow-Up Studies/

58. Evaluation Studies/

59. exp Evaluation Studies as Topic/

60. Lost to Follow-Up/

61. exp Treatment Outcome/

62. exp Morbidity/

63. Feasibility Studies/

64. exp Mortality/

65. Physical Examination/

66. exp Infection Control/

67. Cross Infection/

68. Hospital Information Systems/

69. Comparative Study/

70. exp Survival/

71. Time-to-Treatment/

72. exp Diagnostic Errors/

73. Clinical Decision-Making/

74. Decision Making/

75. exp Quality of Life/

76. morbidity.tw.

77. feasibility.tw.

78. time.tw.

79. mortality.tw.

80. outcome*.tw.

81. conversion.tw.

82. follow-up.tw.

83. followup.tw.

84. decision*.tw.

85. impact.tw.

86. impacts.tw.

87. convert.tw.

88. delay*.tw.

89. adverse effect*.tw.

90. isolation.tw.

91. contact investigation*.tw.

92. default.tw.

93. dropout*.tw.

94. drop-out*.tw.

95. empiric therapy.tw.

96. cure.tw.

97. failure*.tw.

98. relapse*.tw.

99. harm*.tw.

100. prevention.tw.

101. prevented.tw.

102. secondary case*.tw.

103. effectiveness.tw.

104. death*.tw.

105. undertreat*.tw.

106. under treat*.tw.

107. overtreat*.tw.

108. over treat*.tw.

109. adverse event*.tw.

110. adverse outcome*.tw.

111. undesirable effect*.tw.

112. patient centred.tw.

113. patient centered.tw.

114. contact tracing.tw.

115. contact examination*.tw.

116. infection control.tw.

117. cross infection*.tw.

118. treatment fail*.tw.

119. recurrence.tw.

120. "point of care".tw.

121. survival.tw.

122. comparative stud*.tw.

123. "quality of life".tw.

124. qol.tw.

125. hrqol.tw.

126. or/53-125

127. 5 and 52 and 126

128. 127 not (exp Animals/ not exp Humans/)

129. limit 128 to yr="2015 -Current"

***************************************

**Database: Embase.com Classic+Embase**

1 'tuberculosis'/exp

2 'Mycobacterium tuberculosis'/de

3 tuberculosis:ab,ti,kw

4 tb:ab,ti,kw

5 #1 OR #2 OR #3 OR #4

6 'nucleic acid amplification'/de

7 'molecular diagnosis'/de

8 'nucleic acid test*':ab,ti,kw

9 NAAT:ab,ti,kw

10 NAATs:ab,ti,kw

11 NAA:ab,ti,kw

12 'direct amplification':ab,ti,kw

13 'transcription-mediated amplification':ab,ti,kw

14 'RNA amplification*':ab,ti,kw

15 'DNA amplification*':ab,ti,kw

16 'molecular assay*':ab,ti,kw

17 'molecular diagnos*':ab,ti,kw

18 'polymerase chain reaction*':ab,ti,kw

19 PCR:ab,ti,kw

20 PCRs:ab,ti,kw

21 Xpert:ab,ti,kw

22 GeneXpert:ab,ti,kw

23 cepheid:ab,ti,kw

24 "MTB/RIF":ab,ti,kw

25 cobas:ab,ti,kw

26 TaqMan:ab,ti,kw

27 AMTD*:ab,ti,kw

28 MTD:ab,ti,kw

29 'Gen-Probe':ab,ti,kw

30 'ligase chain reaction*':ab,ti,kw

31 LCx:ab,ti,kw

32 'line probe assay*':ab,ti,kw

33 LPA:ab,ti,kw

34 LPAs:ab,ti,kw

35 AMTD*:ab,ti,kw

36 MTBDR*:ab,ti,kw

37 gMTBDR:ab,ti,kw

38 INNO-LiPA:ab,ti,kw

39 ProbeTec:ab,ti,kw

40 loopamp:ab,ti,kw

41 EXPAR:ab,ti,kw

42 LAMP:ab,ti,kw

43 'loop mediated amplification*':ab,ti,kw

44 'Exponential Amplification Reaction*':ab,ti,kw

45 NALF:ab,ti,kw

46 'nucleic acid lateral flow*':ab,ti,kw

47 ('nucleic acid' AND amplification):ab,ti,kw

48 (NAT or NATs):ti

49 (amplified AND 'direct test*'):ab,ti,kw

50 "BD Probe":ab,ti,kw

51 "Tec Direct":ab,ti,kw

52 #6 OR #7 OR #8 OR #9 OR #10 OR #11 OR #12 OR #13 OR #14 OR #15 OR #16 OR #17 OR #18 OR #19 OR #20 OR #21 OR #22 OR #23 OR #24 OR #25 OR #26 OR #27 OR #28 OR #29 OR #30 OR #31 OR #32 OR #33 OR #34 OR #35 OR #36 OR #37 OR #38 OR #39 OR #40 OR #41 OR #42 OR #43 OR #44 OR #45 OR #46 OR #47 OR #48 OR #49 OR #50 OR #51

53 'time'/exp

54 'delayed diagnosis'/de

55 'early diagnosis'/de

56 'comparative effectiveness'/de

57 'evaluation and follow up'/exp

58 'treatment outcome'/exp

59 'morbidity'/exp

60 'feasibility study'/de

61 'mortality'/exp

62 'contact examination'/de

63 'infection control'/exp

64 'cross infection'/de

65 'hospital information system'/de

66 'comparative study'/de

67 'intermethod comparison'/de

68 'survival'/exp

69 'time to treatment'/de

70 'diagnostic error'/exp

71 'clinical decision making'/de

72 'medical decision making'/de

73 'decision making'/de

74 'quality of life'/exp

75 morbidity:ab,ti,kw

76 feasibility:ab,ti,kw

77 time:ab,ti,kw

78 mortality:ab,ti,kw

79 outcome*:ab,ti,kw

80 conversion:ab,ti,kw

81 'follow-up':ab,ti,kw

82 followup:ab,ti,kw

83 decision*:ab,ti,kw

84 impact:ab,ti,kw

85 impacts:ab,ti,kw

86 convert:ab,ti,kw

87 delay*:ab,ti,kw

88 'adverse effect*':ab,ti,kw

89 isolation:ab,ti,kw

90 'contact investigation*':ab,ti,kw

91 default:ab,ti,kw

92 dropout*:ab,ti,kw

93 'drop-out*':ab,ti,kw

94 'empiric therapy':ab,ti,kw

95 cure:ab,ti,kw

96 failure*:ab,ti,kw

97 relapse*:ab,ti,kw

98 harm*:ab,ti,kw

99 prevention:ab,ti,kw

100 prevented:ab,ti,kw

101 'secondary case*':ab,ti,kw

102 effectiveness:ab,ti,kw

103 death*:ab,ti,kw

104 undertreat*:ab,ti,kw

105 'under treat*':ab,ti,kw

106 overtreat*:ab,ti,kw

107 'over treat*':ab,ti,kw

108 'adverse event*':ab,ti,kw

109 'adverse outcome*':ab,ti,kw

110 'undesirable effect*':ab,ti,kw

111 'patient centred':ab,ti,kw

112 'patient centered':ab,ti,kw

113 'contact tracing':ab,ti,kw

114 'contact examination*':ab,ti,kw

115 'infection control':ab,ti,kw

116 'cross infection*':ab,ti,kw

117 'treatment fail*':ab,ti,kw

118 recurrence:ab,ti,kw

119 'point of care':ab,ti,kw

120 survival:ab,ti,kw

121 'comparative stud*':ab,ti,kw

122 'quality of life':ab,ti,kw

123 qol:ab,ti,kw

124 hrqol:ab,ti,kw

125 #53 OR #54 OR #55 OR #56 OR #57 OR #58 OR #59 OR #60 OR #61 OR #62 OR #63 OR #64 OR #65 OR #66 OR #67 OR #68 OR #69 OR #70 OR #71 OR #72 OR #73 OR #74 OR #75 OR #76 OR #77 OR #78 OR #79 OR #80 OR #81 OR #82 OR #83 OR #84 OR #85 OR #86 OR #87 OR #88 OR #89 OR #90 OR #91 OR #92 OR #93 OR #94 OR #95 OR #96 OR #97 OR #98 OR #99 OR #100 OR #101 OR #102 OR #103 OR #104 OR #105 OR #106 OR #107 OR #108 OR #109 OR #110 OR #111 OR #112 OR #113 OR #114 OR #115 OR #116 OR #117 OR #118 OR #119 OR #120 OR #121 OR #122 OR #123 OR #124

126 #5 AND #52 AND #125

127 #126 NOT ('animal'/exp NOT 'human'/exp)

128 #127 AND [1-1-2015]/sd

***************************

**Web of Science**

(TS=(tuberculosis OR tb)) AND (TS=("nucleic acid test*" OR NAAT OR NAATs OR NAA OR "direct amplification" OR "transcription-mediated amplification" OR "RNA amplification*" OR "DNA amplification*" OR "molecular assay*" OR "molecular diagnos*" OR "polymerase chain reaction*" OR PCR OR PCRs OR Xpert OR GeneXpert OR cepheid OR "MTB/RIF" OR AMPLICOR OR cobas OR TaqMan OR AMTD* OR MTD OR "Gen-Probe" OR "ligase chain reaction*" OR LCx OR "line probe assay*" OR LPA OR LPAs OR AMTD* OR MTBDR* OR gMTBDR OR "INNO-LiPA" OR loopamp OR EXPAR OR LAMP OR "loop mediated amplification*" OR "Exponential Amplification Reaction*" OR NALF OR "nucleic acid lateral flow*") OR (TS=("nucleic acid") AND TS=(amplification)) OR TS=(NAT OR NATs) OR (TS=(amplified) AND TS=("direct test*")) OR TS=("BD Probe" OR "Tec Direct")) AND (TS=(morbidity OR feasibility OR time OR mortality OR outcome* OR conversion OR follow-up OR followup OR decision* OR impact OR impacts OR convert OR delay* OR "early diagnos*" OR "adverse effect*" OR isolation OR "contact investigation*" OR default OR dropout* OR "drop-out*" OR "empiric therapy" OR cure OR failure* OR relapse* OR harm* OR prevention OR prevented OR "secondary case*" OR effectiveness OR death* OR undertreat* OR "under treat*" OR overtreat* OR "over treat*" OR "adverse event*" OR "adverse outcome*" OR "undesirable effect*" OR "patient centred" OR "patient centered" OR "contact tracing" OR "contact examination*" OR "infection control" OR "cross infection*" OR "treatment fail*" OR recurrence OR "point of care" OR survival OR "comparative stud*" OR "quality of life" OR qol OR hrqol))

Applied Date Limit: January 1, 2015

********************************

**Global Health Database**

1. exp Mycobacterium tuberculosis/

2. tuberculosis.mp.

3. tb.tw.

4. or/1-3

5. molecular genetics techniques/ and exp diagnosis/

6. dna amplification/

7. rna amplification/

8. exp polymerase chain reaction/

9. ligase chain reaction/

10. nucleic acid test*.tw.

11. NAAT.tw.

12. NAATs.tw.

13. NAA.tw.

14. direct amplification.tw.

15. transcription-mediated amplification.tw.

16. RNA amplification*.tw.

17. DNA amplification*.tw.

18. molecular assay*.tw.

19. molecular diagnos*.tw.

20. polymerase chain reaction*.tw.

21. PCR.tw.

22. PCRs.tw.

23. Xpert.tw.

24. GeneXpert.tw.

25. cepheid.tw.

26. "MTB/RIF".tw.

27. cobas.tw.

28. TaqMan.tw.

29. AMTD*.tw.

30. MTD.tw.

31. Gen-Probe.tw.

32. ligase chain reaction*.tw.

33. LCx.tw.

34. line probe assay*.tw.

35. LPA.tw.

36. LPAs.tw.

37. AMTD*.tw.

38. MTBDR*.tw.

39. gMTBDR.tw.

40. INNO-LiPA.tw.

41. ProbeTec.tw.

42. loopamp.tw.

43. EXPAR.tw.

44. LAMP.tw.

45. loop mediated amplification*.tw.

46. Exponential Amplification Reaction*.tw.

47. NALF.tw.

48. nucleic acid lateral flow*.tw.

49. (nucleic acid and amplification).tw.

50. (NAT or NATs).ti.

51. (amplified and direct test*).tw.

52. BD Probe.tw.

53. Tec Direct.tw.

54. or/5-53

55. exp time/

56. early diagnosis/

57. treatment failure/

58. morbidity/

59. feasibility studies/

60. exp mortality/

61. "causes of death"/

62. exp death/

63. contact tracing/

64. exp disease control/

65. infection control/

66. cross infection/

67. exp survival/

68. screening/

69. decision making/

70. "quality of life"/

71. morbidity.tw.

72. feasibility.tw.

73. time.tw.

74. mortality.tw.

75. outcome*.tw.

76. conversion.tw.

77. follow-up.tw.

78. followup.tw.

79. decision*.tw.

80. impact.tw.

81. impacts.tw.

82. convert.tw.

83. delay*.tw.

84. adverse effect*.tw.

85. isolation.tw.

86. contact investigation*.tw.

87. default.tw.

88. dropout*.tw.

89. drop-out*.tw.

90. empiric therapy.tw.

91. cure.tw.

92. failure*.tw.

93. relapse*.tw.

94. harm*.tw.

95. prevention.tw.

96. prevented.tw.

97. secondary case*.tw.

98. effectiveness.tw.

99. death*.tw.

100. undertreat*.tw.

101. under treat*.tw.

102. overtreat*.tw.

103. over treat*.tw.

104. adverse event*.tw.

105. adverse outcome*.tw.

106. undesirable effect*.tw.

107. patient centred.tw.

108. patient centered.tw.

109. contact tracing.tw.

110. contact examination*.tw.

111. infection control.tw.

112. cross infection*.tw.

113. treatment fail*.tw.

114. recurrence.tw.

115. "point of care".tw.

116. survival.tw.

117. comparative stud*.tw.

118. "quality of life".tw.

119. qol.tw.

120. hrqol.tw.

121. or/55-120

122. 4 and 54 and 121

123. limit 122 to yr="2015 -Current"

Database: Embase Classic+Embase <1947 to 2015 January 29>

Search Strategy:

--------------------------------------------------------------------------------

1 exp tuberculosis/ (232802)

2 Mycobacterium tuberculosis/ (57525)

3 tuberculosis.mp. (265363)

4 tb.tw. (58072)

5 or/1-4 (301560)

6 nucleic acid amplification/ (4872)

7 molecular diagnosis/ (4583)

8 nucleic acid test*.tw. (886)

9 NAAT.tw. (312)

10 NAATs.tw. (208)

11 NAA.tw. (6639)

12 direct amplification.tw. (244)

13 transcription-mediated amplification.tw. (341)

14 RNA amplification*.tw. (629)

15 DNA amplification*.tw. (3916)

16 molecular assay*.tw. (2074)

17 molecular diagnos*.tw. (10596)

18 polymerase chain reaction*.tw. (186836)

19 PCR.tw. (431859)

20 PCRs.tw. (2951)

21 Xpert.tw. (463)

22 GeneXpert.tw. (200)

23 cepheid.tw. (340)

24 "MTB/RIF".tw. (218)

25 cobas.tw. (2822)

26 TaqMan.tw. (13731)

27 AMTD*.tw. (91)

28 MTD.tw. (6672)

29 Gen-Probe.tw. (753)

30 ligase chain reaction*.tw. (536)

31 LCx.tw. (2337)

32 line probe assay*.tw. (625)

33 LPA.tw. (4609)

34 LPAs.tw. (146)

35 AMTD*.tw. (91)

36 MTBDR*.tw. (176)

37 gMTBDR.tw. (1)

38 INNO-LiPA.tw. (580)

39 ProbeTec.tw. (117)

40 loopamp.tw. (12)

41 EXPAR.tw. (10)

42 LAMP.tw. (16871)

43 loop mediated amplification*.tw. (32)

44 Exponential Amplification Reaction*.tw. (9)

45 NALF.tw. (37)

46 nucleic acid lateral flow*.tw. (21)

47 (nucleic acid and amplification).tw. (5106)

48 (NAT or NATs).ti. (678)

49 (amplified and direct test*).tw. (142)

50 BD Probe.tw. (5)

51 Tec Direct.tw. (0)

52 or/6-51 (585569)

53 exp time/ (525789)

54 comparative effectiveness/ (8107)

55 exp "evaluation and follow-up"/ (1457223)

56 exp "treatment outcome"/ (979386)

57 exp morbidity/ (218643)

58 feasibility study/ (49946)

59 exp mortality/ (684720)

60 contact examination/ (2529)

61 exp infection control/ (80445)

62 cross infection/ (22558)

63 hospital information system/ (18032)

64 comparative study/ (743598)

65 intermethod comparison/ (179489)

66 exp survival/ (604552)

67 time to treatment/ (783)

68 exp diagnostic error/ (60382)

69 clinical decision making/ (16127)

70 medical decision making/ (65597)

71 decision making/ (140931)

72 exp "quality of life"/ (254950)

73 morbidity.tw. (327569)

74 feasibility.tw. (126601)

75 time.tw. (2635179)

76 mortality.tw. (637272)

77 outcome*.tw. (1189970)

78 conversion.tw. (164846)

79 follow-up.tw. (842102)

80 followup.tw. (32105)

81 decision*.tw. (270132)

82 impact.tw. (640283)

83 impacts.tw. (54478)

84 convert.tw. (24620)

85 delay*.tw. (437321)

86 adverse effect*.tw. (130415)

87 isolation.tw. (235688)

88 contact investigation*.tw. (572)

89 default.tw. (8909)

90 dropout*.tw. (8173)

91 drop-out*.tw. (6479)

92 empiric therapy.tw. (2227)

93 cure.tw. (94662)

94 failure*.tw. (695553)

95 relapse*.tw. (162955)

96 harm*.tw. (127568)

97 prevention.tw. (465774)

98 prevented.tw. (198315)

99 secondary case*.tw. (1603)

100 effectiveness.tw. (346458)

101 death*.tw. (738817)

102 undertreat*.tw. (5180)

103 under treat*.tw. (11600)

104 overtreat*.tw. (3330)

105 over treat*.tw. (1937)

106 adverse event*.tw. (116356)

107 adverse outcome*.tw. (18732)

108 undesirable effect*.tw. (3368)

109 patient centred.tw. (2978)

110 patient centered.tw. (6705)

111 contact tracing.tw. (1348)

112 contact examination*.tw. (140)

113 infection control.tw. (17400)

114 cross infection*.tw. (2817)

115 treatment fail*.tw. (26267)

116 recurrence.tw. (255869)

117 "point of care".tw. (8801)

118 survival.tw. (787636)

119 comparative stud*.tw. (102549)

120 "quality of life".tw. (206653)

121 qol.tw. (30987)

122 hrqol.tw. (10399)

123 or/53-122 (9669144)

124 5 and 52 and 123 (4402)

125 124 not (animal not human).sh. (4307)
