# Supplementary material for: Impact of molecular diagnostic tests on diagnostic and treatment delays in tuberculosis: a systematic review and meta-analysis
Source: BMC Infect Dis. 2022 Dec 14;22:940. doi: 10.1186/s12879-022-07855-9 (PMC9748908; doi:10.1186/s12879-022-07855-9)
Supplement: Supplementary file 2 — Additional file 2. Funnel plots. Outputs from the analysis of risk of bias. [file 12879_2022_7855_MOESM2_ESM.docx]

Additional File 2: Funnel plots

# Figure S1: Drug-sensitive TB: diagnostic delay


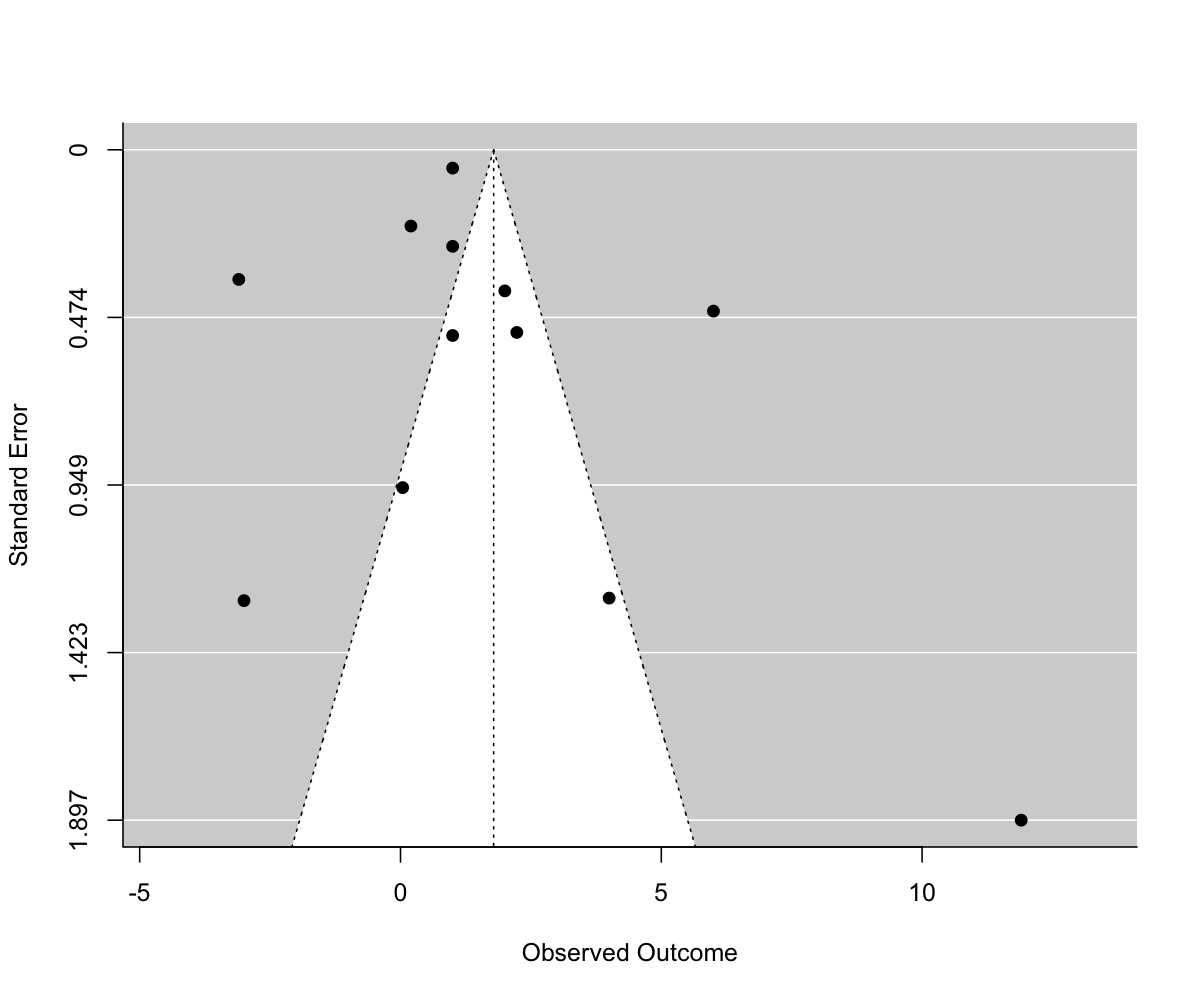


Egger’s test (p = 0.085)

# Figure S2: Drug-sensitive TB: treatment delay


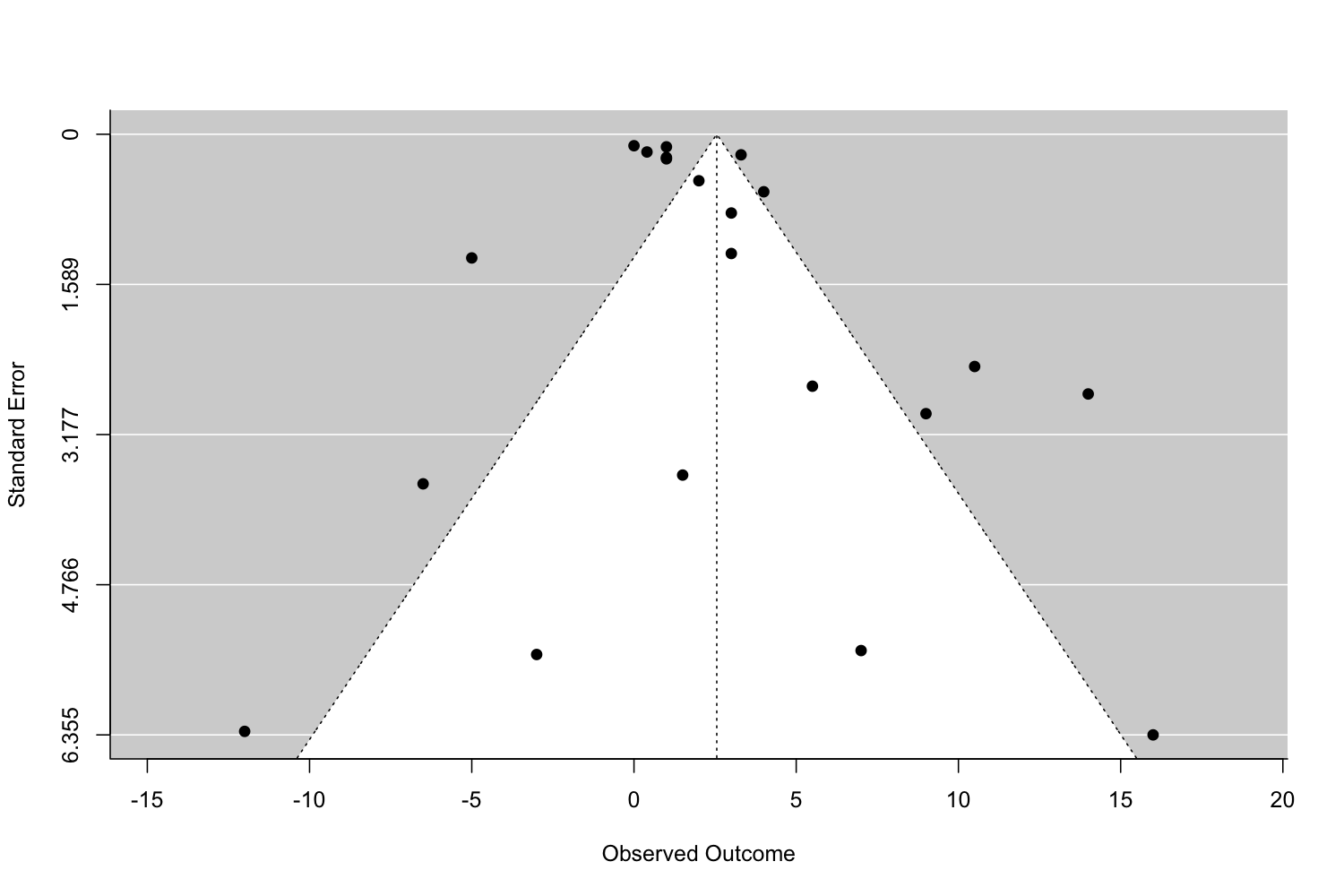


Egger’s test (p = 0.46)

# Figure S3: Drug-resistant TB: diagnostic delay


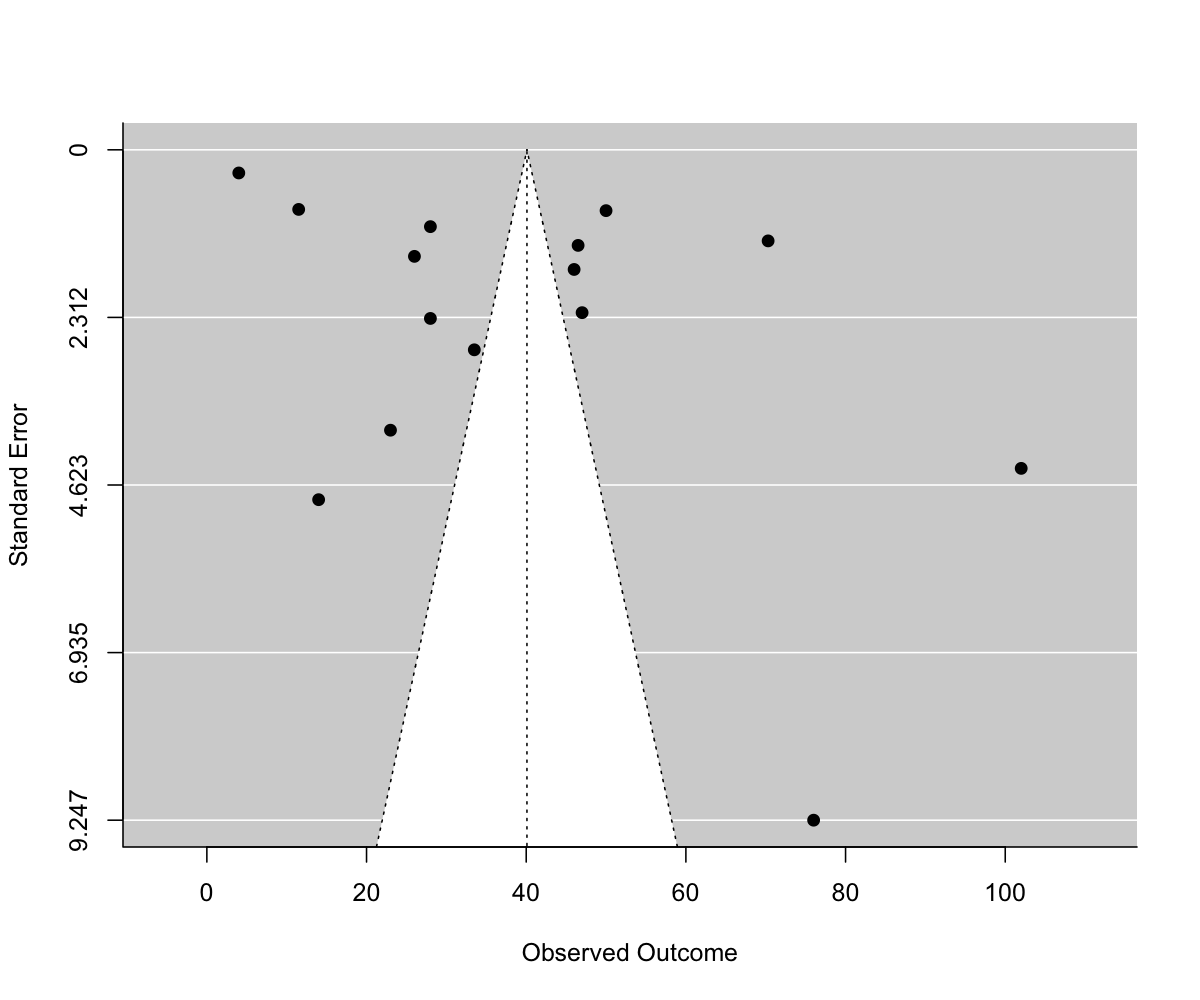


Egger’s test (p = 0.097)

# Figure S4: Drug-resistant TB: treatment delay


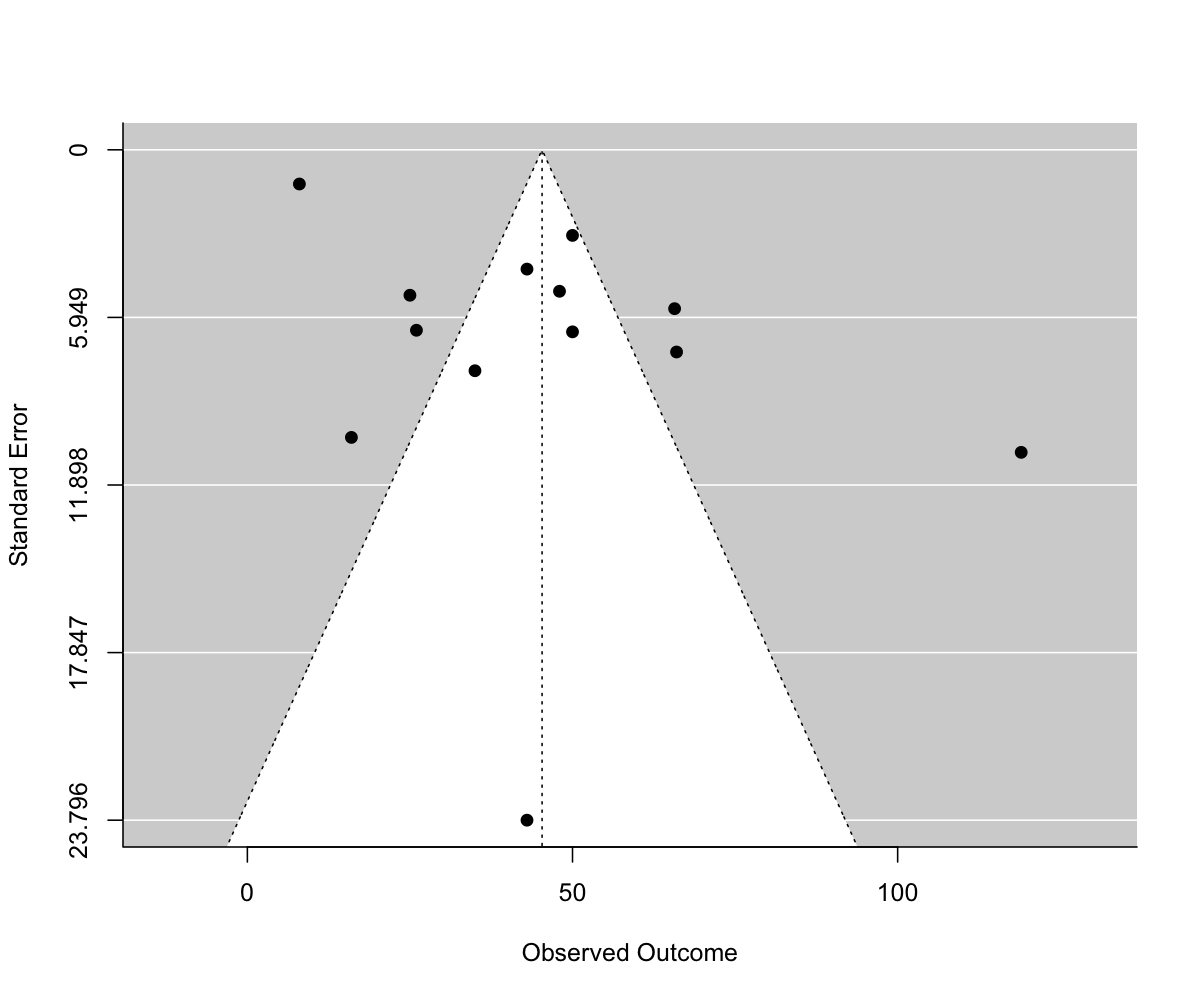


Egger’s test (p = 0.39)
