# Supplementary material for: Impact of molecular diagnostic tests on diagnostic and treatment delays in tuberculosis: a systematic review and meta-analysis
Source: BMC Infect Dis. 2022 Dec 14;22:940. doi: 10.1186/s12879-022-07855-9 (PMC9748908; doi:10.1186/s12879-022-07855-9)
Supplement: Supplementary file 3 — Additional file 3. Data extraction tool. The template for the data extraction tool. [file 12879_2022_7855_MOESM3_ESM.docx]

# Additional File 3: Data extraction tool

| **Final Decision** | Reviewer 1 | Reviewer 2 |
| --- | --- | --- |
| **General information** | | |
| Title |  |  |
| Study ID |  |  |
| Year of Publication |  |  |
| Study start year |  |  |
| Study end year |  |  |
| First author |  |  |
| Corresponding author name |  |  |
| Corresponding author contact details |  |  |
| Funding sources |  |  |
| Country in which the study conducted |  |  |
| Income level of country? (per World Bank definition) |  |  |
| Comment/Explanation (for income level of the country) |  |  |
| BRICS contry? (Brazil, Russia, India, China or South Africa) |  |  |
| WHO region |  |  |
| Index test |  |  |
| MTB-detection: If the study compared additional tests/technologies, please indicate the test(s) |  |  |
| Drug resistance: If the study compared additional tests/technologies, please indicate the test(s) |  |  |
| LPA testing procedure |  |  |
| Implementation Strategy. |  |  |
| Purpose of Index test |  |  |
| Comparator test (Baseline). To what baseline test is time delay measured against? |  |  |
| Comparator test (DST). To what baseline test is time delay measured against? |  |  |
| Author-defined study design (copy relevant section from article; type “none” if none given) |  |  |
| Proportion spontaneously produced sample (format: 0.9 for 90%, 1 for 100%, etc) |  |  |
| **Contextual factors** | | |
| Total number of participants |  |  |
| Further explanation for number of participants |  |  |
| **Epidemiological context** | | |
| HIV prevalence |  |  |
| Proportion of all culture+ that is smear- |  |  |
| Does the study provide primary time-delay results of |  |  |
| Proportion of confirmed TB that is RIF-resistant or MDR-TB |  |  |
| **Quality of Baseline** | | |
| Availability of other diagnostic modalities (e.g. CXR). “unclear” if unclear. |  |  |
| Use of empiric therapy. Proportion of all treated patients that lack microbiological confirmation. (“unclear if unclear; NR for not reported) |  |  |
| **Operational Context** | | |
| Setting in country |  |  |
| Level of healthcare system where patients were managed |  |  |
| Laboratory where samples were tested (Index test). |  |  |
| Comment/ further explanation (Index). |  |  |
| Laboratory where samples were tested (Comparator test). |  |  |
| Comment/ further explanation (Comparator). |  |  |
| Inpatients/ outpatients. |  |  |
| Sample transport index test |  |  |
| Sample transport comparator test |  |  |
| Number lost to follow up (numerator) |  |  |
| Number lost to follow up (denominator) |  |  |
| Comment/explanation for proportion lost to follow up |  |  |
| Other loss to follow up |  |  |
| **Time-Delay Outcomes** | | |
| Defining & Reporting of Time-Delay Outcomes |  |  |
| Was time delay primary outcome of the study? |  |  |
| If ‘no’, what is the primary outcome of the study? |  |  |
| Which of the following time delay components did the author report? (Refer to the operational definition in the protocol. Check all that apply.) |  |  |
| For Authors only reported reduction in time; write in the Comment which time delay among our operational definition the authors reported as 'reduction in time') |  |  |
| Of the reported time delays, were author defined time delays consistent with our operational definition of time delay? (See the table in the protocol) |  |  |
| If not consistent or uncertain, please indicate inconsistencies. |  |  |
| Did the study report time delay for both the index and comparator test? |  |  |
| For which diagnosis did the study report the time delay outcomes? |  |  |
| How was time delay reported? (Median (IQR) or Mean (95% CI), etc.) |  |  |
| **MTB detection** | | |
| **Index test** | | |
| Time delay reported (Index test) – Median/mean |  |  |
| Time delay reported (Index test) – Lower (IQR/95% CI) |  |  |
| Time delay reported (Index test) – Upper (IQR/95% CI) |  |  |
| Time delay reported (Index test) – sample size |  |  |
| **Comparator test** | | |
| Time delay reported (Comparator test) – Median/mean |  |  |
| Time delay reported (Comparator test) – Lower (IQR/95% CI) |  |  |
| Time delay reported (Comparator test) – Upper (IQR/95% CI) |  |  |
| Time delay reported (Comparator test) – sample size |  |  |
| **Third test (if any)** | | |
| Time delay reported (Third test) – Median/mean |  |  |
| Time delay reported (Third test) – Lower (IQR/95% CI) |  |  |
| Time delay reported (Third test) – Upper (IQR/95% CI) |  |  |
| Time delay reported (Third test) – sample size |  |  |
| **(Airborne) isolation time - Index test** | | |
| Time reported (Index test) - Median/mean |  |  |
| Time reported (Index) - Lower (IQR/95% CI) |  |  |
| Time reported (Index) - Upper (IQR/95% CI) |  |  |
| Time reported (Index) - Sample size |  |  |
| **(Airborne) isolation time - Comparator test** | | |
| Time reported (Comparator) - Median/mean | | |
| Time reported (Comparator) - Lower (IQR/95% CI) | | |
| Time reported (Comparator) - Upper (IQR/95% CI) | | |
| Time reported (Comparator) - Sample size | | |
| **Drug susceptibility testing (DST)** | | |
| **Index test** | | |
| Time delay reported (Index test) – Median/mean |  |  |
| Time delay reported (Index test) – Lower (IQR/95% CI) |  |  |
| Time delay reported (Index test) – Upper (IQR/95% CI) |  |  |
| Time delay reported (Index test) – sample size |  |  |
| **Comparator test** | | |
| Time delay reported (Comparator test) – Median/mean |  |  |
| Time delay reported (Comparator test) – Lower (IQR/95% CI) |  |  |
| Time delay reported (Comparator test) – Upper (IQR/95% CI) |  |  |
| Time delay reported (Comparator test) – sample size |  |  |
| **Third test (if any)** | | |
| Time delay reported (Third test) – Median/mean |  |  |
| Time delay reported (Third test) – Lower (IQR/95% CI) |  |  |
| Time delay reported (Third test) – Upper (IQR/95% CI) |  |  |
| Time delay reported (Third test) – sample size |  |  |
| **Therapeutic delay (Time to treatment initiation)** | | |
| **Index test** | | |
| Time delay reported (Index test) – Median/mean |  |  |
| Time delay reported (Index test) – Lower (IQR/95% CI) |  |  |
| Time delay reported (Index test) – Upper (IQR/95% CI) |  |  |
| Time delay reported (Index test) – sample size |  |  |
| **Comparator test** | | |
| Time delay reported (Comparator test) – Median/mean |  |  |
| Time delay reported (Comparator test) – Lower (IQR/95% CI) |  |  |
| Time delay reported (Comparator test) – Upper (IQR/95% CI) |  |  |
| Time delay reported (Comparator test) – sample size |  |  |
| **Third test (if any)** | | |
| Time delay reported (Third test) – Median/mean |  |  |
| Time delay reported (Third test) – Lower (IQR/95% CI) |  |  |
| Time delay reported (Third test) – Upper (IQR/95% CI) |  |  |
| Time delay reported (Third test) – sample size |  |  |
| **Factors (risk) influencing time delays** | | |
| Did the study evaluate risk factors for time delays? |  |  |
| How were risk factors associated time delay evaluated? Describe author’s methodology. |  |  |
| What were the positive factors (risk) were reported in the study (e.g. Reported in relative risk/ odds ratio or other indicators. Statistically significant factors only). |  |  |
| What were the negative factors (risk) were reported in the study (e.g. Reported in relative risk/ odds ratio or other indicators. Statistically significant factors only). |  |  |
| Mode of time-delay report |  |  |
